# Supplementary material for: Modulating ferroptosis sensitivity: environmental and cellular targets within the tumor microenvironment
Source: J Exp Clin Cancer Res. 2024 Jan 13;43:19. doi: 10.1186/s13046-023-02925-5 (PMC10787430; doi:10.1186/s13046-023-02925-5)
Supplement: Supplementary file 1 — Supplementary Material 1 [file 13046_2023_2925_MOESM1_ESM.docx]

**Modulating Ferroptosis Sensitivity: Environmental and Cellular Targets within the Tumor Microenvironment**

Yuze Hua^1^*, Sen Yang^1^*, Yalu Zhang^1, 2^*, Jiayi Li^1^, Mengyi Wang^1^, Palashate·Yeerkenbieke^1, 3^, Quan Liao^1#^, Qiaofei Liu^1#^

1. Department of General Surgery, State Key Laboratory of Complex Severe and Rare Diseases, Peking Union Medical College Hospital, Chinese Academy of Medical Science, and Peking Union Medical College, Beijing 100730, China.

2. Department of General Surgery, Anhui Provincial Hospital, The First Affiliated Hospital of USTC, Division of Life Science and Medicine, University of Science and Technology of China, Hefei, 230027, China.

3. Department of General Surgery, Xinjiang Yili Kazak Autonomous Prefecture Friendship Hospital, Xinjiang, 835099, China.

* Equal contribution

# Corresponding author:

Prof. Qiaofei Liu ([qfliu@aliyun.com](mailto:qfliu@aliyun.com)) and Prof. Quan Liao([lqpumc@126.com](mailto:lqpumc@126.com)), Department of General Surgery, State Key Laboratory of Complex Severe and Rare Diseases, Peking Union Medical College Hospital, Chinese Academy of Medical Sciences & Peking Union Medical College, 1# Shuaifuyuan, Dongcheng District, Beijing 100730, China.

**Abstract:**

Ferroptosis, a novel form of cell death triggered by iron-dependent phospholipid peroxidation, presents significant therapeutic potential across diverse cancer types. Central to cellular metabolism, the metabolic pathways associated with ferroptosis are discernible in both cancerous and immune cells. This review begins by delving into the intricate reciprocal regulation of ferroptosis between cancer and immune cells. It subsequently details how factors within the tumor microenvironment (TME) such as nutrient scarcity, hypoxia, and cellular density modulate ferroptosis sensitivity. We conclude by offering a comprehensive examination of distinct immunophenotypes and environmental and metabolic targets geared towards enhancing ferroptosis responsiveness within the TME. In sum, tailoring precise ferroptosis interventions and combination strategies to suit the unique TME of specific cancers may herald improved patient outcomes.

**Keywords**: Ferroptosis, Cellular metabolism, Tumor Immunity, Tumor Microenvironment

**1. Introduction**

Cancer development and progression are intricately associated with the multifaceted tumor microenvironment (TME). This dynamic milieu is shaped by aberrant environmental conditions and complex cellular interactions [1, 2]. In the TME, cancer cells interact with various immune cells such as MDSCs, Tregs, and M2-type TAMs. These immunosuppressive cells collectively hinder the metabolism of T cells required for recruitment, infiltration, proliferation, and activation [3-5]. Moreover, The TME is characterized by challenging nutrient deprivation, hypoxia, and acidosis, which significantly influences cellular metabolism. Such conditions compromise the function of anti-tumoral T cells and enhance the resistance to therapeutic interventions [6-10]. It is imperative, therefore, to achieve a deeper understanding of the cellular and environmental interplay in terms of metabolic regulation within the TME. Such knowledge can provide insights into tailoring the properties of the TME for improved cancer therapy outcomes [3, 11].

Ferroptosis is a relatively novel form of non-apoptotic regulated cell death characterized by distinct morphological, biochemical, and genetic features [12, 13]. Growing evidence implicates ferroptosis in various pathological conditions and diseases, including tumors, cardiomyopathies, ischemia-reperfusion injuries, and neurodegenerative disorders [14-17]. In the field of oncology, ferroptosis has shown potential therapeutic efficacy for a range of cancer types, notably pancreatic ductal adenocarcinoma (PDAC), ovarian cancer, and melanoma [15, 18-22]. The mechanism that influences ferroptosis sensitivity within the TME remains to be fully elucidated. In this review, the focus will be on understanding the differential sensitivities to ferroptosis in the TME, especially as shaped by its environmental and cellular regulation, to establish precise strategies for ferroptosis-based tumor therapy.

**2. Overview of Ferroptosis**

Ferroptosis, first found in 2012, is characterized by iron-dependent and reactive oxygen species (ROS)-mediated membrane phospholipid containing polyunsaturated fatty acid chains (PUFA-PL) peroxidation, producing excessive PUFA-phospholipid hydroperoxides (PUFA-PLOOH), which leads to membrane rupture and cell death [12, 20]. The synthesis of PUFA-PLOOH relies on two ways, including the enzyme-based mechanism and the non-enzymic mechanism. Massive ROS produced from a non-enzymic Fenton reaction initiated by iron, transported by transferrin receptor. In the presence of iron, PUFA-PLOOH forms alkoxyl phospholipid radical (PLO·), and eventually triggers ferroptosis [23]. ROS plays a vital role in ferroptosis as the promotor of lipid peroxidation. The mitochondria, the major source of ROS, are an influencing factor in ferroptosis sensitivity [24]. Acyl-CoA synthetase long-chain family member 4 (ACSL4) and lysophosphatidylcholine acyltransferase 3 (LPCAT3) promote ferroptosis by enriching the polyunsaturated fatty acids (PUFAs) on the cell membrane lipids [25]. System Xc^-^, also known as SLC7A11, on the cell membrane uptakes Cystine (Cys_2_), reduces it to Cysteine (Cys) by thioredoxin reductase (TrxR), and then synthesizes glutathione (GSH) the primary substance neutralizing ROS as well as forming the glutathione peroxidase 4 (GPX4) as the subunit [26]. GPX4 is the main detoxification enzyme that reduces PLOOH to phosphatidyl alcohol PLOH, thereby inhibiting ferroptosis [27]. Recently, research revealed that Cys directly promoted the synthesis of GPX4 by activating mTOR [28]. TRX system, mainly composed of Thioredoxin (Trx) and TrxR, is an alternative antioxidant system related to ferroptosis [29, 30]. As a co-factor of GPX4, Trx promotes the expression and function of GPX4 [29, 31]. TrxR1 mediates the reduction of Cys_2_ transported by system Xc^-^ into endogenous Cys synergistically with GPX4 [32]. By alternatively promoting system Xc^-^ expression, TrxR1 sustains cell survival after GSH expression is inhibited [32]. A recent study found that the TRX system inhibitors could induce ferroptosis in cancer cells [33]. Coenzymes Q10 (CoQ_10_) is another antioxidant system parallel to GPX4. It is reduced by ferroptosis suppressor protein 1 (FSP1) to a lipophilic radical-trapping antioxidant (RTA) combating ferroptosis [34, 35]. Tetrahydrobiopterin (BH4) biosynthesis essentially and alternatively regulates ferroptosis sensitivity by inhibiting the production of PLOOH after GPX4 inhibition [36]. Dihydroorotate dehydrogenase (DHODH), an enzyme in mitochondria, resists ferroptosis occurring in mitochondria [37]. The systemic molecular mechanisms of ferroptosis have been comprehensively reviewed somewhere else [14, 20, 38]. The schematic diagrams of related regulations are summarized in **Figure 1.**

**3. Environmental Stresses Recalibrate Metabolism within the TME**

3.1 Ferroptosis defense mechanism of T cells

Upon activation, CD4^+^ and CD8^+^ effector T cells require essential GPX4 to avoid ferroptotic cell death [27, 39, 40]. Interestingly, while system Xc^-^ plays a significant role in other cell types, it appears less essential for the proliferation and immunological function of T cells. Indeed, investigations within PDAC models have demonstrated that suppression of system Xc^-^ expression notably curtails tumor growth without compromising the systemic anti-tumor immunity mediated by T cells in vivo. This implies the existence of alternative Cys procurement mechanisms within T cells [41]. In the TME, T cells employ alanine-serine-cysteine transporters (ASCT1 and ASCT2) to assimilate both Cys_2_ and Cys, predominantly from activated antigen-presenting cells (APCs) such as macrophages and dendritic cells (DCs) [41-44]. These APCs, orchestrate the synthesis of both intracellular and extracellular Trx, which facilitates the breakdown of Cys_2_ to Cys for T cell absorption. This process is regulated by the transcription factor, nuclear factor erythroid 2-related factor 2 (NRF2). Notably, the inhibition of NRF2 in T cells is associated with significant downregulation of pivotal molecules including glutamate-cysteine ligase (GCL), system Xc^-^, and GSH synthase [42, 45, 46].

The existence and completeness of a trans-sulfurization pathway in T cells, which would potentially convert methionine to Cys, remains an academic debate. The present study suggests that while such a pathway might be operational, its capacity to satiate the Cys demands of the cell is likely limited [42, 44, 47]. An additional note of interest is the observation that memory T cells exhibit heightened resistance to ferroptosis, a trait possibly attributed to their augmented mitochondrial content, which facilitates enhanced energy production with a concomitant reduction in peroxide generation [48].

3.2 Interplay between ferroptosis and the anti-tumor activity of T cells

Emerging research has elucidated the role of effector T cells in promoting ferroptosis within cancer cells [49-51]. Detailed in vitro and in vivo experiments have revealed a distinct molecular mechanism underpinning this phenomenon: activated CD8^+^ T cells secrete IFN-γ which induces ferroptosis in cancer cells. This is achieved through the binding of IFN-γ to IFN-γ receptor I (IFNGR1) on cancer cells, which subsequently activates the JAK/STAT signaling pathway to inhibit the transcriptional activity of downstream system Xc^-^ [51, 52]. In tandem, CD4^+^ T cells further potentiate the sensitivity of cancer cells to ferroptosis by secreting TNF-α, which binds to TNF-α receptor 1 (TNFR1) on the cancer cells. This interaction results in the inhibition of GSH synthesis and subsequently improves the production of ROS via the activation of nicotinamide adenine dinucleotide phosphate oxidase (NOXs). It is worth noting that the ferroptotic effects induced by this mechanism can be counteracted by the addition of Cys or the use of a TNF-α neutralizing antibody, underscoring the pivotal roles of these molecules in the ferroptotic process [53]. Significantly, the anti-tumor efficacy of IFN-γ appears to be a foundational requirement for the subsequent effects of TNF-α. This is potentially attributed to IFN-γ diminishing the reservoir of GSH within the TME, thereby creating a conducive cellular milieu for TNF-α-mediated effects [54].

3.3 Enhanced resistance to ferroptosis in T regulatory cells (Tregs)

Within the intricate milieu of the tumor microenvironment, Tregs play a pivotal role in modulating immune responses, predominantly deriving their energy from fatty acid oxidation [55]. Emerging evidence suggests that Tregs exhibit an augmented resistance to ferroptosis, potentially attributed to their augmented synthesis and secretion of Trx [56-60]. Upon activation, Tregs appear to bolster their anti-ferroptotic defenses by upregulating GPX4. Interestingly, experiments involving GPX4 knock-out in Tregs have demonstrated that co-stimulation of TCR and CD28 leads to an overproduction of superoxide in the mitochondria, thereby precipitating ferroptosis [61]. IL-1β, along with other pro-inflammatory mediators, is released following Treg ferroptosis. This not only triggers T helper 17 (TH17) responses but also augments the activation of DCs and amplifies the anti-tumor efficacy of antigen-specific CD8^+^ T cells [62-64]. Paradoxically, oxidative stress-induced apoptosis in Tregs has been observed to release significant amounts of adenosine intriguingly undermining the benefits of immune checkpoint blockade (ICB) [65]. All in all, the nuanced interplay between Treg ferroptosis, cytokine release, and immune modulation offers a promising avenue for therapeutic interventions. The selective secretion of IL-1β by ferroptotic Tregs, in particular, emerges as a potential target in advanced cancer treatments.

3.4 Differential sensitivity to ferroptosis in M2-type versus M1-type TAMs

The TME is enriched with various immune cell populations, among which TAMs play a multifaceted role. Notably, the dichotomy between M1 and M2 types of TAMs offers intriguing insights into their contrasting roles and susceptibilities in oncological contexts. M2-type TAMs, which have been recognized as potent facilitators of tumor progression, intriguingly exhibit an enhanced vulnerability to ferroptosis compared to their M1-type counterparts [66]. One of the keystones ensuring the survival of M2-type TAMs in the face of ferroptosis is the pivotal enzyme, GPX4. In stark contrast, M1-type TAMs, encompassing the specialized microglia cells, are equipped with inducible nitric oxide synthase (iNOS). This enzyme is instrumental in producing nitric oxide (NO), a radical molecule endowed with highly reactive chemical properties. As a key modulator of ferroptosis, NO influences the peroxidation of specific lipid moieties, particularly those containing PUFAs such as arachidonic acids (AAs). The ensuing catalytic action, primarily mediated by arachidonic acid lipoxygenase 15 (ALOX-15), fortifies the ferroptosis resistance inherent to M1-type TAMs and microglial cells [67]. The ability of NO to move freely across cellular membranes allows it to protect neighbouring cells from ferroptosis [67, 68]. In contrast, the heightened susceptibility of M2-type TAMs to ferroptosis can be attributed to iNOS deficiency. This distinction presents a tantalizing therapeutic opportunity. Harnessing the proclivity of M2-type TAMs towards ferroptosis could offer a dual advantage in cancer therapeutics: not only annihilating malignant cells but also concurrently mitigating the tumor-promoting effects of M2-type TAMs.

3.5 Dynamic Cys competition between MDSCs and T cells

In the TME, a nuanced interplay exists between MDSCs and tumor-infiltrating T cells, with Cys acting as a pivotal molecule around which their interactions revolve. MDSCs, by their metabolic and functional dynamics, exhibit a pronounced propensity to engender ferroptosis in tumor-infiltrating T cells. This is achieved primarily through their competitive consumption of both Cys_2_ and Cys. Notably, MDSCs actively import Cys_2_ through system Xc^-^, subsequently engaging in its conversion to Cys. Although their import rate for Cys_2_ mirrors that of T cells, a distinguishing feature of MDSCs is their augmented intracellular storage capability. Their reluctance to export Cys back into the TME, coupled with their active uptake of Cys_2_, results in a significant reduction in the extracellular Cys_2_. As a result, T cells face a diminished availability of Cys [47]. Furthermore, cancer-infiltrating MDSCs uniquely express elevated levels of N-acylsphingosine amidohydrolase (ASAH2). This enzyme plays a decisive role in modulating redox dynamics by destabilizing the p53 protein, thereby curbing ROS production and rendering MDSCs resistant to ferroptosis. Targeting ASAH2, by inhibition, instigates ferroptosis in MDSCs. Such a targeted intervention has demonstrated therapeutic potential, evidenced by the augmented activity of tumor-infiltrating cytotoxic T lymphocytes (CTLs) and subsequent attenuation of tumor progression [69].

3.6 Neutrophil ferroptosis and its immunosuppressive effect on anti-tumor immunity

Tumor-associated neutrophils (TANs) within the TME present a dichotomous role, with their function straddling both immune promotion and suppression. Such duality can be attributed to their intrinsic functional plasticity and vast immunological heterogeneity, determinants that collectively shape the clinical trajectory of oncological cases [70]. A subtype, the polymorphonuclear myeloid-derived suppressor cells (PMN-MDSCs)—pathologically hyperactive neutrophils—are susceptible to ferroptosis. Intriguingly, while ferroptotic pathways lead to a reduction in PMN-MDSCs, they concurrently intensify their immunosuppressive phenotypes. This is exemplified by the augmented accumulation of AA-PE and subsequent prostaglandin E2 (PGE2) release, impairing T-cell functions and facilitating tumor progression [71]. In the context of glioblastoma (GBM) pathogenesis, the early phase witnesses the upregulation of granulocyte-colony stimulating factor (G-CSF) among other cytokines, resulting from GBM activity, which orchestrates TAN activation. The ensuing high presence of mature TANs propels GBM cell ferroptosis by ferrying myeloperoxidase (MPO) particles, with neutrophilic signatures, into GBM cells. This process, mediated by N-cadherin, can occur through direct interactions or proximal engagements [72]. Following this, necrotized cancer cells liberate an ensemble of damage-associated molecular patterns (DAMPs), IL-8, IL-6, CCL2, and CXCL1, orchestrating microglial recruitment, thereby fostering tumor invasiveness and culminating in a self-reinforcing loop [73]. This scenario underscores ferroptosis not merely as a cell death pathway but as a critical determinant modulating tumor growth dynamics and invasiveness [74] (**Figure 3**). It is imperative, therefore, to discern the specific roles of ferroptosis in shaping the TME, as this offers prospective therapeutic avenues.

3.7 Enhancing ferroptosis resistance of cancer-associated fibroblasts (CAFs) in cancer cells

CAFs are not mere bystanders in the TME; they serve as crucial facilitators of tumor progression. Recent studies underscore their critical role in modulating the delicate balance of ferroptosis [75]. One of the salient mechanisms by which CAFs bolster cancer cell resilience against ferroptosis is through the provision of vital antioxidants, specifically GSH and Cys. These molecules have been identified as central mediators in the resistance against both platinum-based therapies and ferroptosis-induced cell death. A recent discovery pinpointed a nuanced strategy wherein CAFs secrete exosome-derived miR-522, which post-transcriptionally inhibits the translation of ALOX15 mRNA in gastric cancer cells. ALOX15 plays an important role in the lipid peroxidation process and the accumulation of ROS, which is a hallmark of ferroptosis. By dampening ALOX15 translation, CAFs indirectly shield gastric cancer cells from ferroptotic death [76]. However, this protective aura crafted by CAFs is not unchallenged. CD8^+^ T cells have been observed to counteract the antioxidative strategies of CAFs. They secrete IFN-γ, which, upon binding to its cognate receptor interferon regulatory factor 1 (IRF1) on CAFs, promotes the transcription of gamma-glutamyl transferases (GGT5). GGT5 degrades the extracellular GSH that CAFs might acquire, thereby limiting their antioxidant potential. Further, IFN-γsuppresses system Xc^-^ transcription by activating the JAK/STAT pathway in CAFs. Such suppression culminates in reduced intracellular pools of GSH and Cys, rendering the neighboring cancer cells susceptible to ferroptosis once more [54].

3.8 The role of lipid peroxidation in influencing DCs antigen presentation

DCs serve as the linchpins of immune initiation, especially within the tumor milieu, where Tumor-associated DCs (TADCs) play a quintessential role in igniting and sustaining T-cell-mediated anti-tumor responses. However, the integrity of their function is not unchallenged in the TME. Recent investigations have pointed to a paradoxical relationship between lipid peroxidation, a pivotal component of ferroptosis, and the antigen presentation abilities of TADCs. Excessive inflammation, especially induced by lipopolysaccharide (LPS), has been found to precipitate ferroptotic events in DCs. Moreover, a seminal study delineated that a surge in LPS levels led not just to ferroptosis but concomitantly instigated immune dysregulation in DCs. Delving deeper into the molecular intricacies revealed Sesn2, traditionally known for its role in apoptosis regulation, as a protective entity safeguarding DCs against ferroptosis, potentially orchestrated via the ATF4-CHOP-CHAC1 signaling cascade [77]. However, another layer of complexity is added by 4-hydroxynonenal (4-HNE), a by-product of lipid peroxidation prevalent in the TME. This molecule was discerned to impair TADCs' antigen presentation capabilities. Mechanistic insights divulged that 4-HNE exerts its effects primarily by perpetuating the activation of the X-box binding protein (XBP1) gene, subsequently triggering a cascade of endoplasmic reticulum (ER) stress events. This stress, in turn, culminates in an accumulation of oxidized lipids and a surfeit of ROS, collectively compromising the antigen-presenting capacity of TADCs [78, 79].

Interestingly, the activation dynamics of TADCs also unravel a distinct molecular interplay involving ALOX12 and ALOX15 enzymes. These enzymes are found to actively peroxidize both exogenous sources like low-density lipoproteins (LDL) and endogenous membrane lipids, transmuting them into oxidized phosphatidylcholine (Ox-PC). Notably, this transformation, especially in an ischemia-reperfusion injury model, induces ferroptosis and concomitantly stimulates the antioxidant guardian NRF2, which in synergy with 4-HNE, impedes DC maturation. Moreover, the lipid peroxidation machinery, notably NOX2 located in the endosomal membrane of DCs, facilitates the ingress of tumor-associated antigens into the cytoplasm, a crucial step for efficient cross-presentation to CD8+ T cells [80]. It's imperative to note that M2-TAMs, known for their pro-tumorigenic attributes, also express ALOX12 and ALOX15, raising pertinent questions regarding their potential roles in TAM maturation and polarization dynamics[81-84]. Accordingly, it provides a profound insight into the potential immunosuppressive attributes of TADCs, underscoring the need for targeted therapeutic interventions."

3.9 Natural Kill (NK) cell function regulated by ferroptosis

NK cells have always been recognized for their adeptness in eliminating malignant cells that deftly evade CD8+ T cell surveillance [85]. Within the tumor microenvironment, a specialized subset, Tumor-associated NK (TANK) cells, emerge as pivotal sentinels. Intriguingly, the TME, a hotspot of lipid peroxidation stress, casts a profound influence on these TANK cells. This stress manifests not just as a biochemical change but translates to discernible alterations in the NK cells, most notably, ferroptosis-like cellular morphology and the induction of proteins traditionally associated with ferroptosis. The implications of this lipid peroxidation stress are multifold. A consequential reduction in glycolysis, a crucial metabolic pathway for TANK cell effector functions, dampens their anti-tumor ardor. Yet, nature's intrinsic checks and balances emerge in the form of NRF2, a linchpin in ferroptosis regulation. NRF2 orchestrates a metabolic recalibration in TANK cells, reviving both glucose metabolism and oxidative phosphorylation (OXPHOS), with a pivotal assist from glutamine supplementation [86]. Further delineating the NK cell-ferroptosis nexus, novel therapeutic modalities like CAR-NK cells have entered the limelight. Their ability to induce cancer cell ferroptosis, primarily through the release of IFN-γ which downregulates the SLC3A and SLC7A11 expression, is noteworthy [87]. However, this intricate relationship warrants deeper exploration, as it promises potential breakthroughs in therapeutic applications. Understanding this dynamic could be pivotal in reshaping cancer therapeutics, as schematically presented in **Figure 2**.

3.10 Dynamic modulation of immune response by ferroptotic cancer cells through DAMPs

The intersection of ferroptosis and its potential to induce immunogenic cell death (ICD) remains a contentious issue in current cancer biology research. Although some studies underscore ferroptosis exhibiting hallmark attributes of ICD, the relationship appears to be nuanced and temporally regulated [88]. In the initial stages of ferroptosis, there is a prominent release of DAMPs intrinsically related to ICD. Concomitant with this is an elevated ROS production and endoplasmic reticulum (ER) stress, which further magnify DAMP surface exposure. This amplified DAMP exposure is posited to enhance the CTLs-mediated anti-tumor response, providing a potential avenue for leveraging immune responses in cancer treatment [88-91]. Endometrial carcinoma (EC), especially in its early and low-grade manifestations, offers compelling insights into this dynamic. Here, initial ferroptotic cell phases have been associated with the secretion of ATP and HMGB1, quintessential DAMPs. These molecules are subsequently identified by TAMs and DCs, resulting in their maturation and activation. Once activated, APCs intensify the inflammatory milieu by secreting cytokines like IL-6, potentially enhancing T-cell infiltration into the tumor and thus bolstering the adaptive immune response [92]. However, a pivotal consideration is the dynamic nature of DAMP release. As ferroptosis progresses, the consistent depletion of DAMPs, notably ATP and HMGB1, results in the gradual abrogation of cancer cell immunogenicity. A case in point is calreticulin (CRT), a canonical DAMP. CRT, upon release from apoptotic cells, transforms Ecto-CRT, playing a key role in modulating APC behavior [88, 93]. However, Peter et al recently found that ferroptosis is not a kind of ICD regardless of ferroptosis stages although typical anti-tumor DAMPs are indeed released in this process. Ferroptosis cancer cells reduce DC maturation and cross-presentation of soluble antigens and consequently inhibit immune response.

Some DAMPs released by ferroptotic cancer cells fuel cancer progression [89-91]. These DAMPs are usually produced in the process of ferroptosis and derived from peroxidative byproducts in cancer cells. 1-steaoryl-2-15-HpETE-sn-glycero-3-phosphatidylethanolamine (SAPE-OOH) accumulates on the ferroptotic cancer cell membrane and emits an “eat me” signal upon ferroptosis occurs. Recognized by a kind of pattern recognition receptor (PRR), the toll-like receptor (TLR) 2 of TAMs, SAPE-OOH directly guides them to phagocytize ferroptotic cells [94]. Another recent study revealed that peroxide stress triggers autophagy-dependent ferroptosis of PDAC cells and these cells produce DAMP-like 4-HNE and 8-hydroxy-2′-deoxyguanosine (8-OHG) to accelerate the migration and infiltration of TAMs [95, 96]. TAMs take up exosomes containing KRASG12D protein, a kind of DAMPs released by ferroptotic PDAC cells, through advanced glycosylation end product-specific receptor (AGER) and polarize into the immunosuppressive type by promoting STAT3-dependent fatty acid oxidation [97-99]. 8-OHG and GMP-AMP synthase (cGAS), two by-products of DNA peroxidation damage in cancer cells attract and activate M2-TAMs by mediating stimulator of interferon genes (STING) pathway activation in the stroma. These TAMs pathologically release various cytokines like IL-6 and NOS2 and ultimately promote the occurrence and development of PDAC [96]. Whether these tumor-promoting effects are time-dependent remains unknown.

**4. Metabolism Reprogramming and ferroptosis vulnerability by Environment Stresses in TME**

Ferroptosis is closely associated with cellular redox metabolism and environmental condition. In the TME, cells adjust metabolic pattern and intensity in response to environmental stresses and reprogrammed metabolism is the hallmark of cancers. The competition for nutrients and extreme environmental conditions like acidosis and hypoxia in TME ultimately regulate ferroptosis and accelerate cancer development **(Figure 4)** [100-102].

4.1 Enhancing ferroptosis resistance of cancer by glucose starvation in TME

Within the TME, rapidly proliferating cells, such as malignant cells and activated T cells, predominantly rely on glucose as their primary energy source through heightened aerobic glycolysis [103]. Recent findings have illustrated that cancer cells, in conjunction with myeloid cells, consume the majority of the available glucose in the TME [104]. Notably, the depletion of glucose appears to confer protection against ferroptosis for both cancer cells and immunosuppressive cells, while concurrently attenuating the anti-tumor functionalities of activated T cells within the TME [86, 100]. Intriguingly, a study postulated that under conditions of glucose scarcity, cancer cells adapt by depleting their ATP reserves, allowing them to evade ferroptosis. This energy crisis catalyzes the activation of the AMP-activated protein kinase (AMPK) pathway, which in turn suppresses the synthesis of PUFAs, consequently imparting resistance to ferroptosis [105]. In another pivotal study, it was demonstrated that the uptake of glucose via glucose transporter 1 (GLUT1) potentiates cystine-dependent ferroptosis in PDAC cells. Mechanistically, glucose facilitates glycolysis to promote pyruvate oxidation, TCA cycle and fatty acid synthesis. Glucose deprivation-induced overexpression of pyruvate dehydrogenase kinase 4 (PDK4) subsequently inhibits the tricarboxylic acid (TCA) cycle and lipid peroxidation processes, facilitated by ALOX5 [106]. Of clinical relevance, the efficacy of CD8^+^ T cells against tumors diminishes in a glucose-deprived environment [107]. Conversely, active regulatory T cells (Tregs) appear to flourish under such glucose constraints, preferentially metabolizing lactic acid to fuel processes such as the TCA cycle and gluconeogenesis, thereby sustaining their suppressive activities [108]. Despite the pro-tumor advantages conferred by glucose deprivation, one must not overlook the metabolic vulnerabilities it exposes. Recent insights have elucidated that cancer cells with elevated expression of system Xc^-^ become susceptible under glucose-restricted conditions in the TME. Pertinently, the targeted inhibition of system Xc^-^ can incite ferroptosis in these cancer cells [109].

4.2 Amino acid competition for ferroptosis resistance in the TME

The aggressive proliferation of cancer cells, combined with the aberrant tumor vasculature, leads to a scarcity of Cys_2_, Cys, and Glutamine (Gln) in the TME [110, 111]. This sets off intensified oxidative stress, pushing cells within the TME to intensify their reliance on the GSH-based antioxidant system [112, 113]. Notably, anti-tumor immune cells compete with cancer cells and immunosuppressive cells, for Cys and Cys_2_ [114].

Emerging evidence from PDAC and chronic B lymphocytic leukemia (CLL) investigations suggests that cancer cells employ alternate strategies to circumvent ferroptosis in the TME. Fascinatingly, in vivo tests show that system Xc^-^-KO PDAC cells can still foster tumors in athymic mice [114-116]. A cyclical exchange emerges involving Cys and Cys_2_ between system Xc^-^-KO cancer cells, CAFs, and unaltered cancer cells in the TME. Specifically, CAFs and the native cancer cells internalize Cys_2_, metabolizing it to Cys, which is then recycled back into the TME. Here, system Xc^-^-KO PDAC cells absorb it via alternative transporters like ASCT1, ASCT2, LAT1, and SNATs [116]. Additionally, bone marrow stromal cells (BMSCs), marked by elevated system Xc^-^ expression, have been observed to exude Cys for neighboring CLL cells with heightened demand for GSH and diminished system Xc^-^ expression [117, 118]. To form a comprehensive understanding, it is imperative to consider the presence and distribution of APCs, MDSCs, and CAFs, as they likely influence the Cys supply dynamics between T cells and cancer cells.

Gln, much like glucose, is also a critical energy substrate for immune and cancer cells. Contemporary in vivo studies have accentuated that diverse tumors prioritize exogenous Gln, underlining its irreplaceable metabolic function [104]. Gln serves as a complementary source for the TCA cycle. Transported chiefly via SNAT1 and ASCT2, Gln is processed into Glu by glutaminase (GLS). This Glu then morphs into α-ketoglutarate (α-KG), central to the TCA cycle and fatty acid biosynthesis, in a process known as glutaminolysis [119-121]. Gao et al. illustrates the essential role of glutaminolysis in promoting CDI ferroptosis. Specifically, part of Glu, synthesized via glutaminolysis, is swapped for Cys through system Xc-, amplifying CDI ferroptosis [119, 122, 123]. Subsequent studies reveal that a significant chunk of exogenous Gln, about a third to half, is essential for the Cys exchange via system Xc^-^ [124].

4.3 Reprogrammed lipids metabolism inhibiting anti-tumor immune

Activated and resting T cells both incorporate external lipids essential for their metabolism [125]. Lipid metabolism acts as a regulator for ferroptosis sensitivity and CD36 play a key role in it. Within the TME, the lipid metabolism of CD8^+^ T cells undergoes a transformation, characterized by elevated expression of CD36 and a noticeable shift towards reliance on PUFAs. Both cholesterol and fatty acids amplify the expression of the fatty acid translocase CD36 in tumor-infiltrating CD8^+^ T cells. This, in turn, boosts the uptake and breakdown of PUFAs and compounds rich in PUFAs, such as oxidized low-density lipoproteins (Ox-LDLs) and oxidized phospholipids (Ox-PLs). The outcome of this shift is twofold: the induction of T-cell ferroptosis and a resultant dysfunction [25, 126-132]. In tandem, lipid peroxidation activates P38 kinase, further suppressing T cell function. However, overexpression of GPX4 has shown promise in rescuing these T cells in vivo [132-134]. Strategically targeting CD36 amplifies the infiltration capacity and anti-tumor response of CD8^+^ T cells [61].

Furthermore, immunosuppressive Tregs exhibit metabolic adaptability in the TME through heightened CD36 expression. Tregs with elevated CD36 expression can preserve their mitochondrial health under high oxidative stress. This is achieved via the peroxisome proliferator-activated receptor-β (PPAR-β), which raises levels of nicotinamide adenine dinucleotide (NAD) and the NAD/NADH ratio [61]. Interestingly, CD36 sees substantial expression in HCC cells, which heightens aerobic glycolysis via the PI3K/AKT/mTOR pathway. This results in an increased lactic acid output, further driving cancer progression [135]. Consequently, the presence of lipids and the acidotic nature of the TME foster ferroptosis in effector T cells but shield Tregs from ferroptosis due to heightened CD36 expression. Extended exposure to cholesterol augments intracellular lipid intake and buildup. This process escalates the expression and activity of GPX4, making cancer cells more resilient against ferroptosis and thus fostering cancer cell metastasis [136].

4.4 Acidification stress enabling cancer cells to evade ferroptosis

Cancer cells intensively utilize anaerobic glycolysis, leading to the production and release of substantial amounts of lactic acid, resulting in extracellular acidification [127, 137]. Hepatocellular carcinoma cells (HCCs) efficiently uptake lactic acid through transporters like hydroxycarboxylic acid receptor 1 (HCAR1) and monocarboxylate transporter 1 (MCT1). Elevated lactic acid levels stimulate the AMPK pathway, leading to an increase in monounsaturated fatty acids (MUFAs) in cell membranes and a decline in ACSL4 expression, helping cells resist ferroptosis [138].

This acidic environment bolsters tumor metastasis by sidestepping ferroptosis. In numerous cancers, lymphatic metastasis tends to precede blood metastasis [139, 140]. Cancer cells within lymphatic fluid exhibit greater resilience against ferroptosis than those in the bloodstream [141, 142]. One proposed mechanism is that the high oxidative stress in the blood promotes ferroptosis in cancer cells, while those in lymphatic fluid remain unscathed [143, 144]. Notably, lymphatic fluid has an abundance of oleic acid, a type of MUFA. Cancer cells harness this, adjusting their lipid profile to decrease the proportion of PUFAs, facilitated by fatty acid transport proteins (FATP). This uptake of oleic acid plays a unique role, shielding cancer cells from ferroptosis in an ACSL3-dependent manner. Further, the GSH/GSSG ratio is markedly elevated in lymphatic fluid, thereby reducing oxidative stress experienced by the cancer cells.

4.5 Modulation of ferroptosis resistance in cancer cells and TAMs mediated by hypoxia

Hypoxic environments have been recognized to play crucial roles in modulating ferroptosis resistance in both immune and cancer cells. In malignant mesothelioma (MM) cells, the overexpression of carbonic anhydrase 9 (CA-9) orchestrates iron metabolism, granting these cells a marked resistance to ferroptosis during hypoxic conditions [145]. Such resistance in the context of hypoxia has also been documented in hepatocellular carcinoma (HCC), cervical cancer, and MM [146, 147]. Similarly, peritoneal metastases of gastric cancer (GC), which are inherently characterized by hypoxia, leverage the HIF-1α pathway to counteract ferroptosis. Specifically, HIF-1α upregulates the transcription of the peritoneal metastasis-associated long non-coding RNA (PMAN). This, in turn, bolsters the production of the ELAV Like RNA Binding Protein 1 (ELAVL1), which stabilizes the translation of SLC7A11 mRNA to defend ferroptosis [148]. However, an anomalous observation in renal clear-cell carcinoma (CCC) reveals that hypoxia, counterintuitively, accentuates the susceptibility of these cancer cells to ferroptosis. Here, HIF-2α selectively augments the cancer cell membrane's lipid composition with PUFAs by inducing the hypoxia-inducible lipid droplet-associated protein (HILPDA) gene. This modulation renders the cancer cells more prone to ferroptosis in a GPX4-dependent manner [149]. Furthermore, hypoxic environments are implicated in the anomalous formation of neovasculature and the accumulation of acid. This cumulatively ushers in nutrient scarcity and an acidic milieu, instigating a metabolic shift in lipid and glucose pathways, which indirectly sways the ferroptosis sensitivity of cells[150, 151].

In tumors with relatively higher oxygen concentrations, exemplified by early-stage lung cancer and lung metastases, cancer cells mitigate their ferroptosis susceptibility. A notable mechanism involves the selective overexpression of the N-ethyl-maleimide sensitive fusion protein (NSF1), a synthetase modulating iron homeostasis, and the assimilation of sulfur in Cys for synthesizing iron-sulfur clusters (ISCs), which curtails the cellular iron burden. By suppressing NFS1 expression, a surge in iron starvation response is observed, thereby synergistically amplifying ferroptosis induced by agents like erastin or through Cys deprivation [152].

On the immune front, hypoxia is observed to skew TAMs towards the M2 phenotype, thereby favoring ferroptosis resistance through iron metabolic pathways [153, 154]. Investigations on osteoclasts reveal that under normoxic conditions, bone marrow-derived macrophages (BMDMs) propel ferroptosis through ferritinophagy and an iron-starvation response, the latter of which is potentiated by the receptor activator of NF-κB ligand (RANKL), leading to iron overload. In contrast, hypoxic conditions induce human primary macrophages to elevate ferritin expression patterns (e.g., mitochondrial FTMT and FTH), diminishing free intracellular iron and shielding them from ferroptosis. At the molecular level, HIFs directly stifle NCOA4 transcription while simultaneously amplifying the JNK pathway, leading to the production of miR-6862-5p which inhibits NCOA4 translation. This intricately modulated pathway culminates in NCOA4 binding to FTMT, inhibiting ferritinophagy , a specialized autophagic process that triggers ferroptosis by the degradation of ferritin [155-157]. Additionally, hypoxic environments bolster HIF-1α expression in macrophages, thereby inhibiting iron-starvation response, which ultimately attenuates their ferroptosis sensitivity [158]. HIF-2α has been also discerned to elevate the expression of ferroportin (FPN), which depletes intracellular iron content, effectively stymieing ferroptosis [159]. Conclusively, the polarization of TAMs into the M2 phenotype and their evasion from ferroptosis is intricately mediated by hypoxia.

**4.6 Cellular interaction and density as a modulator of ferroptosis resistance in neoplasm**

In the intricate milieu of solid tumors, intercellular adhesion, facilitated through E-cadherin, emerges as a central mediator that orchestrates resistance to ferroptosis. This is achieved predominantly via the modulation of the Hippo signaling cascade [160]. Subsequently, this pathway exerts an inhibitory influence on its downstream effector, Yes-associated protein 1 (YAP). The resultant suppression curtails the expression of pivotal proteins such as transferrin receptor 1 (TFRC) and ACSL4, both paramount in determining cellular susceptibility to ferroptosis across in vivo and in vitro paradigms [160]. As another key effector of Hippo pathway, the activity and expression of the PDZ-binding motif (TAZ) is also repressed by escalated cellular density in renal cell carcinoma (RCC) and ovarian cancer (OVCA). TAZ repression has been implicated in reduction of ferroptosis sensitivity, achieved via the inhibition of EMP1-NADPH Oxidase 4 (NOX4) nexus [74, 160-162]. Such repression also precipitates a decline in the production of angiopoietin receptor 4 (ANGPTL4), culminating in attenuated activity of NADPH oxidase 2 (NOX2) and concomitantly ferroptotic susceptibility [163]. Taken together, these underscore the pivotal role of cellular interaction and density in amplifying the resistance to ferroptosis in cancer cells.

**5. Targeting Immunophenotype and Environment Stress to Sensitize Ferroptosis**

The inherent heterogeneity of the TME and the concomitant overlap in metabolic processes governing ferroptosis among immune and neoplastic cells present formidable challenges in optimizing ferroptosis-centric therapeutic modalities [164, 165]. Notably, the manifestation of ferroptosis resistance not only undermines the direct efficacy of ferroptosis inducers but also limits the potential of combinatorial therapeutic strategies. Thus, a ferroptosis combined strategic approach, rooted in the specific environmental, metabolic, and immune characteristics, may offer a tailored mechanism to enhance ferroptotic susceptibility exclusively in cancer cells.

5.1 Immunophenotypic stratification for ferroptosis therapeutics in TME.

The intricate heterogeneity of the TME, along with the shared metabolic dependencies of immune cells and cancer cells on ferroptosis pathways, underscores the complexities of ferroptosis therapy [164, 165]. Integrative approaches, particularly combining immune checkpoint blockade (ICB) with ferroptosis-augmenting agents such as system Xc^-^ inhibitors, Cys modulators, and GPX4 blockers, have showcased promising anti-tumor potential [88, 89, 91, 166]. Yet, the therapeutic suitability hinges crucially on specific immunophenotypes of TME, delineated by precise evaluation criteria. It is imperative to understand that immunophenotyping assesses the spatial distribution, diversity, and density of immune cells within TME landscapes. Based on the dual parameters of tumor immunogenicity and immune cell infiltration, we can broadly classify immunophenotypes into three archetypes: cold, hot, and altered tumors [167]. (Refer to **Table 1** for comprehensive details).

Hot Tumors

Typically marked by high microsatellite instability (MSI-H), these tumors inherently promote robust infiltration of T-cells within the TME core [168]. Considering the heightened susceptibility of activated T cells to ferroptosis, hot tumors may not be prime candidates for initial ferroptosis induction [39]. A deeper dive into the metabolic milieu reveals nuanced interplay, especially around Cys dynamics between cancer cells and T cells, compounded by subsequent competitive interactions with MDSCs. As such, a meticulous evaluation of the spatial distribution and metabolic activity of APCs and MDSCs in juxtaposition to T cells could provide vital predictive insights for ferroptosis therapeutic trajectories [41, 42, 56]. Notably, while hot tumors exhibit a generally favorable response trajectory to immunotherapy, the emergence of therapeutic resistance remains unresolved [169]. This resistance is engendered by a myriad of intrinsic and adaptive immunosuppressive shifts that evolve with dynamic TME alterations, such as PD-L1 overexpression, selective recruitment of immunosuppressive cell phenotypes, and the eventual T cell functional exhaustion [170, 171]. Given this backdrop, the adjunctive use of ferroptosis inducers might offer a therapeutic advantage, particularly as dedifferentiated cancer cell phenotypes, with their accentuated ferroptotic sensitivity, come into focus, where GPX4 emerges as a pivotal mediator [74, 115, 172, 173]. Researchers recently in melanoma model found that ferroptosis is positively associated with immunotherapy and cancer with high ferroptosis activity tends to have a hot TME [174]. Researchers recently also found that ferrptosis-related genes (FRG)-based high-risk score is positively correlated with enrichment of ICB-related positive signatures in the GBM, which possibly predicts ferroptosis treatment combined with immunotherapy [175].

Cold Tumors

Distinctly characterized by a relative lack of central inflammation, these tumors pose a clinical conundrum to elevate CTL infiltration, thereby amplifying the immune response [168, 176]. In this scenario, ferroptosis not only offers therapeutic potential but also serves as an immunological catalyst. Specifically, DAMPs exuded from ferroptotic cancer cells can elicit pronounced immunogenicity, in turn driving the recruitment of APCs and T cells to invoke a tailored adaptive immune response [88, 92, 94, 177]. However, it's crucial to elucidate the temporal kinetics of DAMP release, aiming to determine the optimal therapeutic temporal window.

Altered Tumors

This category encompasses both immune-exclusive and immune-suppressed phenotypes. The former manifests barriers hindering immune cell infiltration, whereas the latter presents a TME saturated with immunosuppressive entities — notably Tregs, MDSCs, and M2-type TAMs — that impair the intrinsic anti-tumor efficacy of immune cells [168, 178, 179]. These immunosupressive cells sensitive to ferroptosis. ICB combined with ferroptosis therapy may have the most clinical benefit for the immunosuppressive type and reshape the immunotype more senstive to immunotherapy. In immune-exclusive phenotypic landscapes, a salient feature is the peripheral T cell localization, barred from accessing the tumor core. This restrictive pattern begs further scrutiny, especially within the framework of ferroptosis and the potential modulatory influence of DAMPs on immune infiltration dynamics. Reasons for the infiltrative barrier of T cells include the lack of chemokines like C-X-C motif ligands (CXCLs) and C-C motif ligands (CCLs), deregulation of extracellular matrix proteins, abnormal vasculature, and hypoxia [180-182]. It appears that this type of TME rarely benefits from ferroptosis-based treatment. Whether some DAMPs released by ferroptosis cancer cells play an alternative role in inducing immune cell infiltration remains obscure. Ferroptosis and its ability to reshape the physical barrier need to be further investigated in the future.

5.2 Leveraging specific metabolic phenotypes of cancer with environmental stressors for ferroptosis sensitization

Tumor-infiltrating cancer cells often manifest distinct metabolic phenotypes that render them more susceptible to ferroptosis. For instance, clear cell renal cell carcinoma (ccRCC) is distinguished by its high lipid and glycogen content, which provides a unique therapeutic vulnerability [142]. A seminal study has elucidated that Von Hippel-Lindau (VHL)-deficient ccRCC, which features characteristic lipid and glycogen-laden cytoplasmic deposits, circumvents ferroptosis via the upregulation of the adipokine chemerin. This upregulation serves a dual role, aiding both autocrine and paracrine signaling of hypoxia-inducible factor (HIF)-2α and krueppel-like factor 6 (KLF6). On a molecular level, chemerin serves to stabilize lipid oxidation and fatty acid oxidation (FAO) equilibrium within the cancer cells. Additionally, it amplifies the expression of HIF-1α and HIF-2α in an autocrine manner, underscoring its multifaceted role in modulating the metabolic landscape of tumors [183]. Furthermore, basal-like breast cancers (BLBC), exhibit specific metabolic shifts favoring transsulfuration. When the TME is starved of Cys_2_ and Cys, cancer cells activate the GCN2-ATF4 axis, bolstering the expression of CTH and CBS, two enzymes vital for the transsulfuration pathway to produce Cys. This metabolic phenotype is highly sensitive to Cys levels [184][185]. The luminal androgen receptor (LAR) subtype of TNBC, a specific metabolic subtype characterized by the increasing expression of oxidized phosphatidylethanolamines and glutathione metabolism, especially GPX4, is hypersensitive to ferroptosis induced by GPX4 inhibitors [186]. Recent findings have illuminated a particular subtype of small cell lung cancer (SCLC) that, while resistant to ferroptosis, displays an addiction to the TRX system. Remarkably, inhibitory interventions targeting TRX render this subtype conspicuously susceptible to ferroptosis [187]. In a similar vein, melanomas that undergo epithelial-to-mesenchymal transition (EMT) exhibit an increased sensitivity to ferroptosis. However, this characteristic is juxtaposed against their inherent resistance to immunotherapies, highlighting the intricate interplay of metabolic and immune landscapes within tumor microenvironments [142].

5.3 Integrative therapeutic strategies by navigating the environmental complexities of the TME

Environmental intricacies within the TME, including hypoxic conditions, nutrient scarcity, and lactic acid accrual, not only bolster neoplastic resistance to ferroptosis but concomitantly exacerbate T cell vulnerability thereto [129, 130, 145, 146]. Consequently, strategies aimed at reconfiguring these TME constraints present intriguing therapeutic avenues, as outlined in **Table 2**.

Due to the glucose starvation, cancer cells exhibit a nuanced metabolic interdependence on both Gln and Cys, situating the system Xc^-^ at a pivotal juncture between nutrient reliance and ferroptosis defense.

A research by Goji et al. highlights that, under glucose-starved conditions in vitro, the additional Cys_2_ uptake via system Xc^-^ swiftly triggers NADPH exhaustion, ROS generation, and ensuing cell death in glioblastoma cells [188]. These revelations spotlight a potential therapeutic avenue: destabilizing the balance between Gln and Cys could potentially thwart cancer cell growth [109]. Some investigations in vivo have demonstrated that solitary Gln blockade not only orchestrates neoplastic cell demise but also markedly augments T cell anti-tumoral activity and ameliorates conditions of hypoxia, acidosis, and nutrient paucity [104, 189, 190]. Conjoint therapeutic regimens encompassing Gln and system Xc^-^ blockades emerge as prospective interventions. Study indicates that cancer cells with an upregulated system Xc- display an increased Gln dependence, though glucose can somewhat alleviate this dependency [189]. This suggests that anti-tumor effects mediated by Gln blockade may amplify on tumor cells with high system Xc expression. Intriguingly, tumors harboring KRAS mutations, exemplified by pancreatic neoplasms, resort to micropinocytosis for extracellular protein uptake, subsequently catabolizing these into Gln [191, 192]. Tumors with MYC oncogenic aberrations exhibit a pronounced Gln metabolic proclivity, postulating its potential utility as a therapeutic biomarker [189, 193].

Acidosis in TME instigates a metabolic paradigm shift, predominantly steered by HIF-1α, transitioning from aerobic glycolysis towards glutamine and lipid metabolism for both energy production and intermediate biosynthesis. Such acidic vicinities, concomitant with hypoxia and metabolite build-up like lactic acid, necessitate therapeutic consideration [194]. Both HIFs and CD36 emerge as pivotal therapeutic fulcrums. Cumulative data in vivo and in vitro authenticate the roles of both HIF-2α and HIF-1α in potentiating CD36 expression, consequently amplifying fatty acid assimilation in hypoxic cells [195, 196]. CD36 inhibition not only disrupts Tregs mitochondrial functionality but also augments ferroptotic sensitization in neoplasms through lactic acid attenuation, while HIFs inhibition prevents the pro-tumor polarization of TAMs, simultaneously reducing neoplastic ferroptotic resistance. Conjoint targeting of CD36 and HIFs offers therapeutic promise.

Moreover, the positive correlation between neoplastic cell interaction and density and ferroptosis resistance suggests that the TME's cellular composition could serve as an indicative parameter for gauging ferroptosis resilience and the clinical efficacy of ferroptosis-centric therapeutics. In this context, the potential exploitation of blockers, such as E-cadherin and the Hippo signaling cascade, remains an area of academic and therapeutic interest.

5.4 Cellular regulation of ferroptosis defensive mechanism in the TME

In cancers predicated on GPX4 dependency, Cys availability emerges as a pivotal determinant of neoplastic resistance to ferroptosis. Within the TME, rigorous competition ensues between cancer cells and T cells for Cys acquisition [41, 45]. The complex cellular conditions within the TME wields a substantial influence on the sensitivity to ferroptosis. Considering the overarching influence of MDSCs, CAFs and M2-TAMs in protecting cancer cells but sensitizing T cells to ferroptosis, particularly in malignancies such as PDAC that are replete with these cellular subsets, integrative strategies that concurrently target these cellular entities and induce cancer cell ferroptosis appear cogent. Additionally, M2-TAMs fortify HIFs expression, which fosters T-cell susceptibility to ferroptosis while concurrently imparting ferroptotic resilience of neoplastic cells. It is noteworthy that DAMPs, when liberated by ferroptotic M2-TAMs, potentiate the immune response of T cells, [87, 197]. An area that warrants deeper academic exploration pertains to the TRX antioxidant system. Historically, its significance might have been somewhat overshadowed. Such insights, while nascent, underscore the profound therapeutic potential inherent in unraveling the complexities of cellular interactions and dependencies within the TME.

**6. Comprehensive Immunotherapy, Chemotherapy and Radiotherapy Treatments with Ferroptosis**

6.1 Immunotherapy treatment with ferroptosis in clinical

Researchers have made plenty of beneficial explorations in the combination of immunotherapy and ferroptosis treatment. In the LAR subtype of TNBC, GPX4 inhibitors enhance the anti-tumor immunity. The combination of GPX4 inhibitor and PD-1 blockade is more efficient than monotherapy in vivo [186]. In glioma, where ferroptosis is the main programmed cell death (PCD), ferroptosis contributed to immunosuppressive TME mediated by TAMs, which leads to malignant progression and poor prognosis. Blocking ferroptosis is thought to be an efficient method to improve the efficacy of ICB [198]. Researchers in melanoma developed a ferroptosis score (FPS) model based on 32 ferroptosis-related genes to predict the outcome of cancer. Then, they found that high-FPS may predict more active tumor immune microenvironment and better efficacy of ICB such as PD-1 blockade [174]. Fan et al. found that BEBT-908, an inhibitor targeting PI3K and HDAC, induced immunogenic ferroptotic cell death of cancer cells and enhanced ICB therapy by upregulating MHC class I and activating IFN-γ signal [199]. Statin promotes immunotherapy by promoting ferroptosis of NSCLC cancer cells and inhibiting PD-L1 expression [200]. FSP1 inhibition greatly promoted ferroptosis of cancer cells and infiltration of immune cells like DCs, macrophages, and T cells. iFSP1 synergistic with immunotherapy significantly reduced the burden of HCC in vivo [201]. Researchers found inflammation-associated ferroptosis (IAF) biomarkers positively predicts the PD-L1 expression, MSI, TMB and ICB response rate [202]. Short-time starvation of methionine promotes ferroptosis by stimulation of CHAC1 transcription synergistically with CD8+ T cells and enhances ICB therapy. Exogenous Methionine intermittent starvation, system xc- and PD-1 blockade combination treatment have a profound anti-tumor efficacy [203]. Ferritin light chain (FTL) facilitates cancer cell ferroptosis in GBM to polarize TAMs into the M2-type contributing to pro-tumor TME. Inhibition of FTL is a promising strategy to sensitize GBM to PD-1 blockade [204].

6.2 Integrating ferroptosis with chemotherapeutic strategies

Chemotherapy remains a pivotal therapeutic modality for a plethora of solid malignancies, yet the specter of chemoresistance invariably poses a significant impediment [205]. Intriguingly, a discernible nexus has been identified between cisplatin resistance and ferroptosis resistance within the tumor milieu. Malignant cells that manifest cisplatin resistance concomitantly exhibit upregulation of genes affiliated with ferroptosis resistance [206-208]. In this context, cisplatin-resistant neoplastic cells overexpress the Wnt pathway membrane receptor, frizzled homolog 7 (FZD7), which subsequently activates TP63, culminating in augmented GPX4 expression [209, 210]. A myriad of cancer phenotypes evolves cisplatin resistance by elevating the expression of system Xc^-^ and preserving elevated GSH concentrations [211-213]. Recent investigations underscore that cisplatin can indeed incite ferroptosis in cancer cells, highlighting the potential role of chemotherapy as a pivotal trigger for ferroptosis [214, 215]. Drug-resistant malignant cells, in tandem with adjacent stromal constituents, might engender a protective cocoon, enabling vulnerable cancerous cells to elude both ferroptosis and chemotherapy. Notably, CAFs have been implicated in curtailing cisplatin accumulation, thereby promoting resistance to both chemotherapy and ferroptosis by proffering GSH and cysteine to ovarian cancer (OVCA) cells [54]. Further, the miR-4443, found to be amplified in cisplatin-resistant non-small cell lung cancer (NSCLC) cells, is conveyed to sensitive cells via exosomes, thereby amplifying FSP1 expression, pivotal for averting ferroptosis. In contrast, IFN-γ, secreted by CD8^+^ T cells, diminishes system Xc^-^ in malignant cells, thereby mitigating cisplatin resistance through the JAK/STAT signaling cascade [54]. The intricate orchestration between cisplatin resistance mechanisms and the ferroptosis pathway cannot be overstated [215]. Poignantly, co-administration of GPX4 and system Xc^-^ inhibitors alongside cisplatin has surmounted chemotherapy resistance, thereby magnifying the anti-neoplastic response in diverse tumors [214, 216, 217].

The resistance to the chemotherapeutic agent Temozolomide (TMZ), primarily deployed for gliomas, is also intricately tethered to ferroptosis resistance, engendered by elevated system Xc^-^ and GSH expression, which concurrently magnify TMZ sensitivity [218, 219]. Furthermore, malignant cells modulate intracellular iron homeostasis via lipocalin-2, thereby obviating ferroptosis and instigating resistance to 5-fluorouracil [220]. Such chemoresistant phenotypes strikingly mirror ferroptosis resistance patterns, especially concerning canonical targets like GPX4, system Xc^-^, FSP1, and NRF2. Consequently, profiling these ferroptosis targets in chemoresistant neoplasms emerges as an imperious necessity. However, the adaptive response to chemotherapy encompasses the induction of the MTDH gene, which amplifies the mesenchymal phenotype of neoplasms, bolstering drug resistance. Nevertheless, diminishing GPX4 and system Xc^-^ expression can augment susceptibility to ferroptosis [221]. Ferroptosis resistance surfaces as a novel rationale underpinning post-chemotherapy tumor resurgence.

For PDAC patients, surgical intervention coupled with adjuvant multi-drug chemotherapy remains the bulwark for enhancing survival. Gemcitabine resistance, inherent in PDAC, continues to perplex clinicians [222]. Recent elucidations intimate that ferroptosis resistance, spearheaded by system Xc^-^hyperexpression, is conjoined with gemcitabine resistance [223, 224]. Intriguingly, system Xc^-^ inhibition in PDAC cells engenders GSH depletion in vitro, precipitating ferroptosis and resuscitating sensitivity to both gemcitabine and cisplatin [115].

The profound interplay between chemoresistance and ferroptosis resistance in neoplasms necessitates re-envisioning therapeutic paradigms. The resurgence of tumors post-chemotherapy can be attributed, at least partially, to ferroptosis resistance. This accentuates the theoretical viability of amalgamated treatment regimens. In this milieu, modulating the TME and metabolic targets to exclusively amplify the ferroptosis sensitivity in malignant cells might emerge as the lynchpin in transcending the barriers of chemoresistance.

6.3 Ferroptosis synergy in radiotherapeutic strategies

Radiotherapy (RT) operates by leveraging ionizing radiation (IR) to catalyze the generation of ROS and free radicals, thereby inflicting damage upon DNA and cellular membranes [225]. Recent revelations suggest that ferroptosis plays a pivotal role as a novel mechanism underlying RT-induced oncogenic cell death [226, 227]. A notable phenotype is that cancer cells demonstrating radioresistance also manifest a concomitant resistance to ferroptosis [227, 228]. Specifically, lung cancers with kelch-like ECH-associated protein 1 (KEAP1) mutations exhibit resistance to ferroptosis and radiotherapy. This is mediated via the augmented transcription of CoQ/FSP1 under the auspices of NRF2. Critically, targeting the CoQ/FSP1 axis can precipitate ferroptosis in KEAP1-deficient lung cancers, thus bolstering their radiosensitivity [229]. This process appears to be intertwined with the surge in ROS and ACSL4 production. However, tumors harboring P53 mutations provide an intriguing counter-narrative. Within this milieu, RT augments the expression of the P53 gene, countering the ferroptosis resistance elicited by system Xc^-^ upregulation and GSH synthesis, leading to enhanced radiosensitivity [230].

The amplification of RT-induced ferroptosis can synergistically potentiate the immune response. From a mechanistic vantage, the radiation-induced bystander effect (RIBE) manifests in RT-afflicted cells through the liberation of radiation-tinged microparticles (RT-MPs). This instigates ICD within proximal cancer cells, predominantly via ferroptosis. Such ferroptotic cells lure an enhanced infiltration of TAMs. These TAMs undergo a transformative polarization towards an anti-tumoral phenotype, guided by the activation of the JAK/STAT and MAPK pathways. Importantly, these TAMs augment the expression of PD-L1 in neoplastic cells through the endocytic uptake of RT-MPs, which markedly enhances immunotherapeutic outcomes [231]. Additionally, activated CD8+ T cells bolster the radiotherapeutic effect via ferroptosis. The tandem actions of IFN-γ derived from CD8+ T cells and the activation of the ataxia-telangiectasia mutated (ATM) gene by radiotherapy synergistically inhibit SCL7A11 expression, optimizing therapeutic outcomes [232, 233].

Yet, hypoxia in the TME can induce hypoxia-inducible factors (HIFs), creating barriers to both radiotherapy and ferroptosis [151]. Consequently, system Xc^-^ and GPX4 witness an adaptive overexpression within cancer cells, offering a sanctuary against radiotherapy. Therefore, ferroptosis inducers can provide a synergistic boon to radiotherapeutic endeavors [226, 234]. Given the profound mechanistic interlinkages between ferroptosis and radiation therapy, factors catalyzing ferroptosis resistance emerge as critical players in shaping radiotherapeutic outcomes. The efficacy of radiotherapy can be substantially amplified by judiciously coupling it with agents that sensitize malignant cells to ferroptosis.

**7. Conclusions and Perspectives**

The overlap of ferroptosis metabolic pathways within both neoplastic and immune cells presents considerable challenges to the specificity and potency of ferroptosis therapeutics. Worse still, the TME seemingly protects malignant cells against ferroptosis while concurrently predisposing T-cells to ferroptosis. To overcome this, a bifurcated approach is worth considering: systematic characterization of the TME in cellular and environmental variables and immunophenotypes to determine its amenability to ferroptosis; subsequent strategic manipulation of salient environmental attributes, such as hypoxia, acidosis, and nutrient dynamics, to alter cellular metabolism. This metabolic recalibration can engender differential sensitivities to ferroptosis, facilitating targeted ablation of malignant and immunosuppressive cells and rescue of T cells. Ferroptotic cells bolster the immune response by releasing DAMPs, notably ATP and HMGB1. Notably, this therapeutic strategy, underscored by its self-reinforcing mechanisms, holds the potential for synergistic integration with established therapeutic modalities, including immunotherapy, radiotherapy, and chemotherapy.

However, notwithstanding its conceptual appeal, this therapeutic paradigm has inherent challenges. A salient concern is the nebulous understanding of how environmental variables modulate ferroptotic sensitivities across varied cell types. Current research endeavors, while illuminative, are predominantly univariate in design, potentially obfuscating the nuanced interactions in the actual TME. Although TIME has been categorized into 'hot', 'cold', and 'altered' states, a holistic classification predicated on environmental variables related to ferroptosis is conspicuously absent. Consequently, an in-depth exploration of these variables is imperative for accurately gauging metabolic vulnerabilities, thereby facilitating nuanced biomarker and therapeutic target identification. Another formidable challenge lies in the intrinsic heterogeneity and dynamism of TME. This heterogeneity is manifest not just across tumor types, but intriguingly, within individuals and even disparate regions of a singular tumor. The inherent metabolic heterogeneity within tumors, characterized by varied metabolic rates and differential hypoxic regions requires meticulous attention [235]. Hence, the quest for an optimal therapeutic window – wherein neoplastic cells are maximally ferroptosis-sensitive and T cells most ferroptosis-resilient – remains paramount. Thus, the universally applicable biomarkers reliably gauging ferroptosis efficacy across varied TME landscapes is paramount. Moreover, the adaptive metamorphosis of the TME significantly fosters resistance not merely to ferroptosis but also other therapeutics such as radiotherapy, chemotherapy, and immunotherapy [76, 236-238]. This underscores the necessity for both proactive characterization and iterative monitoring of the TME throughout therapeutic interventions. Lastly, no efficient and safe drugs targeting ferroptosis in the clinic. While some agents like Artesunate, Sorafenib, and Cisplatin, have shown promise through their dual functionalities, the transition from bench to bedside remains fraught with challenges [239-241]. Factors such as specificity, sensitivity, the quest for spatial-temporal biomarkers, and the identification of clinically viable drugs emerge as critical, yet unresolved conundrums in the realm of ferroptosis therapy.

**Abbreviations**

| 4-HNE | 4-hydroxynonenal |
| --- | --- |
| 8-OHG | 8-hydroxy-2′-deoxyguanosine |
| α-KG | α-ketoglutarate |
| AA | arachidonic acid |
| ACSL4 | acyl-CoA synthetase long-chain family member 4 |
| AGER | advanced glycosylation end product-specific receptor |
| ALOX-15 | arachidonic acid lipoxygenase 15 |
| AMPK | AMP-activated protein kinase |
| ANGPTL4 | angiopoietin receptor 4 |
| APCs | antigen presentation cells |
| ASAH2 | N-acylsphingosine amidohydrolase |
| ASCT | alanine-serine-cysteine transporter |
| ATM | ataxia-Telangiectasia Mutated |
| BH4 | tetrahvdrobiopterin |
| CAFs | cancer-associated fibroblasts |
| CBS | cystathionine β-synthase |
| CCC | clear-cell carcinoma |
| ccRCC | clear cell renal cell carcinoma |
| CDI | cysteine-deprivation-induced |
| cGAS | GMP-AMP synthase |
| CLL | chronic B lymphocytic leukemia |
| CoQ10 | coenzymes Q10 |
| CRT | calreticulin |
| CRT | calreticulin |
| CTH | cystathionine γ-lyase |
| CTX | cyclophosphamide |
| Cys | cysteine |
| Cys2 | cystine |
| DAMPs | damage-associated molecular patterns |
| DCs | dendritic cells |
| DHODH | dihydroorotate dehydrogenase |
| EC | endometrial cancer |
| ECM | extracellular matrix |
| ELAVL1 | peritoneal metastasis associated long noncoding RNA (PMAN) to improve ELAV Like RNA Binding Protein 1 |
| EMP1-NOX4 | epithelial membrane protein 1-NADPH oxidase 4 |
| ER | endoplasmic reticulum |
| FPN | ferroportin |
| FSP1 | ferroptosis suppressor protein 1 |
| FTH | Ferritin heavy (chain) |
| FTMT | mitochondrial ferritin |
| GBM | glioblastoma |
| GC | gastric cancer |
| GCL | glutamate-cysteine ligase |
| G-CSF | granulocyte-colony stimulating factor |
| GGT5 | gamma-glutamyl transferases |
| Gln | Glutamine |
| GLS | Glutaminase |
| GPX4 | glutathione peroxidase 4 |
| GSH | Glutathione |
| HCAR1 | hydroxycarboxylic acid receptor 1 |
| HCC | hepatocellular carcinoma |
| HIFs | hypoxia-inducible factors |
| HILPDA | hypoxia-inducible lipid droplet-associated protein |
| HO-1 | heme oxygenase-1 |
| ICB | immune checkpoint blockade |
| ICD | immunogenic cell death |
| IG | immunogenicity |
| IL-4il | interleukin-4-induced1 |
| ILCs | innate lymphoid cells |
| iNOS | inducible nitric oxide synthase |
| LDL | low-density lipoproteins |
| L-Glu | L-glutamic acid |
| L-Glu | L-glutamic acid |
| MCT1 | monocarboxylate transporter 1 |
| MDSCs | myeloid-derived suppressor cells |
| MMP | mitochondrial membrane potential |
| MPO | myeloperoxidase |
| mROS | mitochondrial ROS |
| MSI-H | high microsatellite instability |
| NAD+/ Coenzyme I | nicotinamide adenine dinucleotide |
| NCOA4 | nuclear receptor coactivator 4 |
| NK cells | natural killer cells |
| NOXs | nicotinamide adenine dinucleotide phosphate oxidase |
| ox-PC | oxidize phosphatidylcholine |
| Ox-PE | oxidized phosphatidylethanolamine |
| PDAC | pancreatic ductal adenocarcinoma |
| PDK4 | pyruvate dehydrogenase kinase 4 |
| PLOOH | phospholipid hydroperoxides |
| PPAR-β | peroxisome proliferator-activated receptor-β |
| PRRs | pattern recognition receptors |
| PUFA | polyunsaturated fatty acids |
| PUFA-PL | polyunsaturated fatty acids-phospholipid |
| PUFAs | polyunsaturated fatty acids |
| RANKL | receptor activator of nuclear factor kappa-B ligand |
| RIBE | radiation-induced bystander effect |
| ROS | reactive oxygen species |
| RT | radiation therapy |
| RTA | radical-trapping antioxidant |
| RT-MPs | radiation therapy-microparticles |
| SAPE-OOH | 1-steaoryl-2-15-HpETE-sn-glycero-3-phosphatidylethanolamine |
| SCD1 | stearoyl-CoA desaturase-1 |
| SNAT1 | sodium-coupled neutral amino acid transporter 1 |
| STING | stimulator of interferon genes |
| TAA | tumor-associated antigens |
| TADCs | tumor-associated dendritic cells |
| TAMs | tumor-associated macrophages |
| ta-NKs | tumor-associated NK cells |
| TANs | tumor-associated neutrophils |
| TAZ | PDZ-binding motif |
| TCA | tricarboxylic acid cycle |
| TFRC | transferrin receptor 1 |
| TLRs | toll-like receptors |
| TME | tumor microenvironment |
| TMZ | temozolomide |
| TNFR1 | TNF-α receptor 1 |
| Trf1 | transferrin receptor 1 |
| TrxR | thioredoxin reductase |
| TXN or TRX | thioredoxin |
| TXNRD1 | thioredoxin reductase1 |
| XBP1 | X-box binding protein |

BMSCs bone marrow stromal cells

**Declarations**

**Ethics approval and consent to participate**

Not applicable.

**Consent for publication**

All authors agreed the publication.

**Availability of data and material**

Not applicable.

**Competing interests**

The authors declare no competing interests.

**Funding**

This work was supported by the National Natural Science Foundation of China (82373230,82373289,82172765), National High-Level Hospital Clinical Research Funding (2022-PUMCH-D-001), CAMS Innovation Fund for Medical Sciences (CIFMS, 2021-I2M-1-002).

**Author Contribution**

YZH, YLZ, and SY draft the manuscript. JYL, MYW, and PY provided advice on the draft. QFL (Qiaofei Liu) and QL (Quan Liao) revised the manuscript. All authors reviewed and approved the final version.

**Acknowledgements**

Not applicable.

**Reference：**

1. Maman, S. and I.P. Witz, *A history of exploring cancer in context.* Nat Rev Cancer, 2018. **18**(6): p. 359-376.

2. Quail, D.F. and J.A. Joyce, *Microenvironmental regulation of tumor progression and metastasis.* Nat Med, 2013. **19**(11): p. 1423-37.

3. Joyce, J.A. and D.T. Fearon, *T cell exclusion, immune privilege, and the tumor microenvironment.* Science (New York, N.Y.), 2015. **348**(6230): p. 74-80.

4. Salmon, H., et al., *Matrix architecture defines the preferential localization and migration of T cells into the stroma of human lung tumors.* The Journal of clinical investigation, 2012. **122**(3): p. 899-910.

5. Borst, J., et al., *CD4 T cell help in cancer immunology and immunotherapy.* Nature reviews. Immunology, 2018. **18**(10): p. 635-647.

6. O'Neill, L.A.J., R.J. Kishton, and J. Rathmell, *A guide to immunometabolism for immunologists.* Nature reviews. Immunology, 2016. **16**(9): p. 553-565.

7. Buck, M.D., et al., *Metabolic Instruction of Immunity.* Cell, 2017. **169**(4): p. 570-586.

8. Vander Heiden, M.G. and R.J. DeBerardinis, *Understanding the Intersections between Metabolism and Cancer Biology.* Cell, 2017. **168**(4): p. 657-669.

9. Kaymak, I., et al., *Immunometabolic Interplay in the Tumor Microenvironment.* Cancer cell, 2021. **39**(1): p. 28-37.

10. Li, X., et al., *Navigating metabolic pathways to enhance antitumour immunity and immunotherapy.* Nature reviews. Clinical oncology, 2019. **16**(7): p. 425-441.

11. Whiteside, T.L., *The tumor microenvironment and its role in promoting tumor growth.* Oncogene, 2008. **27**(45): p. 5904-5912.

12. Dixon, S.J., et al., *Ferroptosis: an iron-dependent form of nonapoptotic cell death.* Cell, 2012. **149**(5): p. 1060-1072.

13. Hadian, K. and B.R. Stockwell, *SnapShot: Ferroptosis.* Cell, 2020. **181**(5).

14. Jiang, X., B.R. Stockwell, and M. Conrad, *Ferroptosis: mechanisms, biology and role in disease.* Nat Rev Mol Cell Biol, 2021. **22**(4): p. 266-282.

15. Fang, X., et al., *Ferroptosis as a target for protection against cardiomyopathy.* Proceedings of the National Academy of Sciences of the United States of America, 2019. **116**(7): p. 2672-2680.

16. Fang, X., et al., *Loss of Cardiac Ferritin H Facilitates Cardiomyopathy via Slc7a11-Mediated Ferroptosis.* Circ Res, 2020. **127**(4): p. 486-501.

17. Li, Y., et al., *Ischemia-induced ACSL4 activation contributes to ferroptosis-mediated tissue injury in intestinal ischemia/reperfusion.* Cell Death Differ, 2019. **26**(11): p. 2284-2299.

18. Tonnus, W. and A. Linkermann, *The in vivo evidence for regulated necrosis.* Immunological reviews, 2017. **277**(1): p. 128-149.

19. Conrad, M., et al., *Regulated necrosis: disease relevance and therapeutic opportunities.* Nature reviews. Drug discovery, 2016. **15**(5): p. 348-366.

20. Stockwell, B.R., et al., *Ferroptosis: A Regulated Cell Death Nexus Linking Metabolism, Redox Biology, and Disease.* Cell, 2017. **171**(2): p. 273-285.

21. Guiney, S.J., et al., *Ferroptosis and cell death mechanisms in Parkinson's disease.* Neurochemistry international, 2017. **104**: p. 34-48.

22. Linkermann, A., et al., *Synchronized renal tubular cell death involves ferroptosis.* Proceedings of the National Academy of Sciences of the United States of America, 2014. **111**(47): p. 16836-16841.

23. Hassannia, B., P. Vandenabeele, and T. Vanden Berghe, *Targeting Ferroptosis to Iron Out Cancer.* Cancer Cell, 2019. **35**(6): p. 830-849.

24. Gao, M., et al., *Role of Mitochondria in Ferroptosis.* Molecular cell, 2019. **73**(2).

25. Doll, S., et al., *ACSL4 dictates ferroptosis sensitivity by shaping cellular lipid composition.* Nature chemical biology, 2017. **13**(1): p. 91-98.

26. Muri, J. and M. Kopf, *Redox regulation of immunometabolism.* Nature reviews. Immunology, 2021. **21**(6): p. 363-381.

27. Yang, W.S., et al., *Regulation of ferroptotic cancer cell death by GPX4.* Cell, 2014. **156**(1-2): p. 317-331.

28. Zhang, Y., et al., *mTORC1 couples cyst(e)ine availability with GPX4 protein synthesis and ferroptosis regulation.* Nature communications, 2021. **12**(1): p. 1589.

29. Zheng, J. and M. Conrad, *The Metabolic Underpinnings of Ferroptosis.* Cell metabolism, 2020. **32**(6): p. 920-937.

30. Zhang, J., et al., *Targeting the Thioredoxin System for Cancer Therapy.* Trends Pharmacol Sci, 2017. **38**(9): p. 794-808.

31. Bai, L., et al., *Thioredoxin-1 Rescues MPP/MPTP-Induced Ferroptosis by Increasing Glutathione Peroxidase 4.* Molecular neurobiology, 2021. **58**(7): p. 3187-3197.

32. Mandal, P.K., et al., *System x(c)- and thioredoxin reductase 1 cooperatively rescue glutathione deficiency.* The Journal of biological chemistry, 2010. **285**(29): p. 22244-22253.

33. Llabani, E., et al., *Diverse compounds from pleuromutilin lead to a thioredoxin inhibitor and inducer of ferroptosis.* Nature chemistry, 2019. **11**(6): p. 521-532.

34. Doll, S., et al., *FSP1 is a glutathione-independent ferroptosis suppressor.* Nature, 2019. **575**(7784): p. 693-698.

35. Bersuker, K., et al., *The CoQ oxidoreductase FSP1 acts parallel to GPX4 to inhibit ferroptosis.* Nature, 2019. **575**(7784): p. 688-692.

36. Soula, M., et al., *Metabolic determinants of cancer cell sensitivity to canonical ferroptosis inducers.* Nature chemical biology, 2020. **16**(12): p. 1351-1360.

37. Mao, C., et al., *DHODH-mediated ferroptosis defence is a targetable vulnerability in cancer.* Nature, 2021. **593**(7860): p. 586-590.

38. Tang, D., et al., *Ferroptosis: molecular mechanisms and health implications.* Cell Res, 2021. **31**(2): p. 107-125.

39. MacKenzie, K.D., et al., *Bistable expression of CsgD in Salmonella enterica serovar Typhimurium connects virulence to persistence.* Infection and immunity, 2015. **83**(6): p. 2312-2326.

40. Suthanthiran, M., et al., *Glutathione regulates activation-dependent DNA synthesis in highly purified normal human T lymphocytes stimulated via the CD2 and CD3 antigens.* Proceedings of the National Academy of Sciences of the United States of America, 1990. **87**(9): p. 3343-3347.

41. Arensman, M.D., et al., *Cystine-glutamate antiporter xCT deficiency suppresses tumor growth while preserving antitumor immunity.* Proceedings of the National Academy of Sciences of the United States of America, 2019. **116**(19): p. 9533-9542.

42. Levring, T.B., et al., *Activated human CD4+ T cells express transporters for both cysteine and cystine.* Scientific reports, 2012. **2**: p. 266.

43. Siska, P.J., et al., *Fluorescence-based measurement of cystine uptake through xCT shows requirement for ROS detoxification in activated lymphocytes.* Journal of immunological methods, 2016. **438**: p. 51-58.

44. Garg, S.K., et al., *Differential dependence on cysteine from transsulfuration versus transport during T cell activation.* Antioxidants & redox signaling, 2011. **15**(1): p. 39-47.

45. Angelini, G., et al., *Antigen-presenting dendritic cells provide the reducing extracellular microenvironment required for T lymphocyte activation.* Proceedings of the National Academy of Sciences of the United States of America, 2002. **99**(3): p. 1491-1496.

46. Gmünder, H., et al., *Macrophages regulate intracellular glutathione levels of lymphocytes. Evidence for an immunoregulatory role of cysteine.* Cellular immunology, 1990. **129**(1): p. 32-46.

47. Srivastava, M.K., et al., *Myeloid-derived suppressor cells inhibit T-cell activation by depleting cystine and cysteine.* Cancer research, 2010. **70**(1): p. 68-77.

48. van der Windt, G.J.W., et al., *Mitochondrial respiratory capacity is a critical regulator of CD8+ T cell memory development.* Immunity, 2012. **36**(1): p. 68-78.

49. Barry, M. and R.C. Bleackley, *Cytotoxic T lymphocytes: all roads lead to death.* Nature reviews. Immunology, 2002. **2**(6): p. 401-409.

50. Golstein, P. and G.M. Griffiths, *An early history of T cell-mediated cytotoxicity.* Nature reviews. Immunology, 2018. **18**(8): p. 527-535.

51. Wang, W., et al., *CD8 T cells regulate tumour ferroptosis during cancer immunotherapy.* Nature, 2019. **569**(7755): p. 270-274.

52. Kong, R., et al., *IFNγ-mediated repression of system xc drives vulnerability to induced ferroptosis in hepatocellular carcinoma cells.* Journal of leukocyte biology, 2021. **110**(2): p. 301-314.

53. Habtetsion, T., et al., *Alteration of Tumor Metabolism by CD4+ T Cells Leads to TNF-α-Dependent Intensification of Oxidative Stress and Tumor Cell Death.* Cell metabolism, 2018. **28**(2).

54. Wang, W., et al., *Effector T Cells Abrogate Stroma-Mediated Chemoresistance in Ovarian Cancer.* Cell, 2016. **165**(5): p. 1092-1105.

55. Michalek, R.D., et al., *Cutting edge: distinct glycolytic and lipid oxidative metabolic programs are essential for effector and regulatory CD4+ T cell subsets.* J Immunol, 2011. **186**(6): p. 3299-303.

56. Matsushita, M., et al., *T cell lipid peroxidation induces ferroptosis and prevents immunity to infection.* The Journal of experimental medicine, 2015. **212**(4): p. 555-568.

57. Mougiakakos, D., et al., *Increased thioredoxin-1 production in human naturally occurring regulatory T cells confers enhanced tolerance to oxidative stress.* Blood, 2011. **117**(3): p. 857-861.

58. Drijvers, J.M., et al., *Pharmacologic Screening Identifies Metabolic Vulnerabilities of CD8 T Cells.* Cancer immunology research, 2021. **9**(2): p. 184-199.

59. Curiel, T.J., et al., *Specific recruitment of regulatory T cells in ovarian carcinoma fosters immune privilege and predicts reduced survival.* Nature medicine, 2004. **10**(9): p. 942-949.

60. Zou, W., *Regulatory T cells, tumour immunity and immunotherapy.* Nature reviews. Immunology, 2006. **6**(4): p. 295-307.

61. Wang, H., et al., *CD36-mediated metabolic adaptation supports regulatory T cell survival and function in tumors.* Nature immunology, 2020. **21**(3): p. 298-308.

62. Ben-Sasson, S.Z., et al., *IL-1 enhances expansion, effector function, tissue localization, and memory response of antigen-specific CD8 T cells.* The Journal of experimental medicine, 2013. **210**(3): p. 491-502.

63. Fotaki, G., et al., *Pro-inflammatory allogeneic DCs promote activation of bystander immune cells and thereby license antigen-specific T-cell responses.* Oncoimmunology, 2018. **7**(3): p. e1395126.

64. Zhivaki, D., et al., *Inflammasomes within Hyperactive Murine Dendritic Cells Stimulate Long-Lived T Cell-Mediated Anti-tumor Immunity.* Cell reports, 2020. **33**(7): p. 108381.

65. Maj, T., et al., *Oxidative stress controls regulatory T cell apoptosis and suppressor activity and PD-L1-blockade resistance in tumor.* Nature immunology, 2017. **18**(12): p. 1332-1341.

66. Piattini, F., et al., *Differential sensitivity of inflammatory macrophages and alternatively activated macrophages to ferroptosis.* European journal of immunology, 2021.

67. Kapralov, A.A., et al., *Redox lipid reprogramming commands susceptibility of macrophages and microglia to ferroptotic death.* Nature chemical biology, 2020. **16**(3): p. 278-290.

68. Mikulska-Ruminska, K., et al., *NO Represses the Oxygenation of Arachidonoyl PE by 15LOX/PEBP1: Mechanism and Role in Ferroptosis.* International journal of molecular sciences, 2021. **22**(10).

69. Zhu, H., et al., *Asah2 Represses the p53-Hmox1 Axis to Protect Myeloid-Derived Suppressor Cells from Ferroptosis.* Journal of immunology (Baltimore, Md. : 1950), 2021. **206**(6): p. 1395-1404.

70. Shaul, M.E. and Z.G. Fridlender, *Tumour-associated neutrophils in patients with cancer.* Nature reviews. Clinical oncology, 2019. **16**(10): p. 601-620.

71. Kim, R., et al., *Ferroptosis of tumour neutrophils causes immune suppression in cancer.* Nature, 2022. **612**(7939): p. 338-346.

72. Sagiv, J.Y., et al., *Phenotypic diversity and plasticity in circulating neutrophil subpopulations in cancer.* Cell reports, 2015. **10**(4): p. 562-573.

73. Christofides, A., M. Kosmopoulos, and C. Piperi, *Pathophysiological mechanisms regulated by cytokines in gliomas.* Cytokine, 2015. **71**(2): p. 377-384.

74. Yee, P.P., et al., *Neutrophil-induced ferroptosis promotes tumor necrosis in glioblastoma progression.* Nature communications, 2020. **11**(1): p. 5424.

75. Sahai, E., et al., *A framework for advancing our understanding of cancer-associated fibroblasts.* Nature reviews. Cancer, 2020. **20**(3): p. 174-186.

76. Zhang, H., et al., *CAF secreted miR-522 suppresses ferroptosis and promotes acquired chemo-resistance in gastric cancer.* Molecular cancer, 2020. **19**(1): p. 43.

77. Li, J.Y., et al., *Sestrin2 protects dendrite cells against ferroptosis induced by sepsis.* Cell Death Dis, 2021. **12**(9): p. 834.

78. Herber, D.L., et al., *Lipid accumulation and dendritic cell dysfunction in cancer.* Nature medicine, 2010. **16**(8): p. 880-886.

79. Cubillos-Ruiz, J.R., et al., *ER Stress Sensor XBP1 Controls Anti-tumor Immunity by Disrupting Dendritic Cell Homeostasis.* Cell, 2015. **161**(7): p. 1527-1538.

80. Dingjan, I., et al., *Lipid peroxidation causes endosomal antigen release for cross-presentation.* Scientific reports, 2016. **6**: p. 22064.

81. Rothe, T., et al., *12/15-Lipoxygenase-mediated enzymatic lipid oxidation regulates DC maturation and function.* The Journal of clinical investigation, 2015. **125**(5): p. 1944-1954.

82. Al-Huseini, L.M.A., et al., *Nuclear factor-erythroid 2 (NF-E2) p45-related factor-2 (Nrf2) modulates dendritic cell immune function through regulation of p38 MAPK-cAMP-responsive element binding protein/activating transcription factor 1 signaling.* The Journal of biological chemistry, 2013. **288**(31): p. 22281-22288.

83. Williams, M.A., et al., *Disruption of the transcription factor Nrf2 promotes pro-oxidative dendritic cells that stimulate Th2-like immunoresponsiveness upon activation by ambient particulate matter.* Journal of immunology (Baltimore, Md. : 1950), 2008. **181**(7): p. 4545-4559.

84. Stamenkovic, A., et al., *Oxidized phosphatidylcholines trigger ferroptosis in cardiomyocytes during ischemia-reperfusion injury.* Am J Physiol Heart Circ Physiol, 2021. **320**(3): p. H1170-h1184.

85. Shimasaki, N., A. Jain, and D. Campana, *NK cells for cancer immunotherapy.* Nat Rev Drug Discov, 2020. **19**(3): p. 200-218.

86. Poznanski, S.M., et al., *Metabolic flexibility determines human NK cell functional fate in the tumor microenvironment.* Cell metabolism, 2021. **33**(6).

87. Wu, L., et al., *The establishment of polypeptide PSMA-targeted chimeric antigen receptor-engineered natural killer cells for castration-resistant prostate cancer and the induction of ferroptosis-related cell death.* Cancer Commun (Lond), 2022. **42**(8): p. 768-783.

88. Efimova, I., et al., *Vaccination with early ferroptotic cancer cells induces efficient antitumor immunity.* Journal for immunotherapy of cancer, 2020. **8**(2).

89. Galluzzi, L., et al., *Consensus guidelines for the definition, detection and interpretation of immunogenic cell death.* Journal for immunotherapy of cancer, 2020. **8**(1).

90. Krysko, D.V., et al., *Immunogenic cell death and DAMPs in cancer therapy.* Nature reviews. Cancer, 2012. **12**(12): p. 860-875.

91. Kroemer, G., et al., *Immunogenic cell death in cancer therapy.* Annual review of immunology, 2013. **31**: p. 51-72.

92. López-Janeiro, Á., et al., *Proteomic Analysis of Low-Grade, Early-Stage Endometrial Carcinoma Reveals New Dysregulated Pathways Associated with Cell Death and Cell Signaling.* Cancers, 2021. **13**(4).

93. Obeid, M., et al., *Calreticulin exposure is required for the immunogenicity of gamma-irradiation and UVC light-induced apoptosis.* Cell death and differentiation, 2007. **14**(10): p. 1848-1850.

94. Luo, X., et al., *Oxygenated phosphatidylethanolamine navigates phagocytosis of ferroptotic cells by interacting with TLR2.* Cell death and differentiation, 2021. **28**(6): p. 1971-1989.

95. Zhou, B., et al., *Ferroptosis is a type of autophagy-dependent cell death.* Seminars in cancer biology, 2020. **66**.

96. Dai, E., et al., *Ferroptotic damage promotes pancreatic tumorigenesis through a TMEM173/STING-dependent DNA sensor pathway.* Nature communications, 2020. **11**(1): p. 6339.

97. Kang, R., et al., *RAGE is essential for oncogenic KRAS-mediated hypoxic signaling in pancreatic cancer.* Cell death & disease, 2014. **5**: p. e1480.

98. Dai, E., et al., *Autophagy-dependent ferroptosis drives tumor-associated macrophage polarization via release and uptake of oncogenic KRAS protein.* Autophagy, 2020. **16**(11): p. 2069-2083.

99. Mohapatra, S., et al., *Non-coding RNAs regulation of macrophage polarization in cancer.* Molecular cancer, 2021. **20**(1): p. 24.

100. Chang, C.-H., et al., *Metabolic Competition in the Tumor Microenvironment Is a Driver of Cancer Progression.* Cell, 2015. **162**(6): p. 1229-1241.

101. Zhao, E., et al., *Cancer mediates effector T cell dysfunction by targeting microRNAs and EZH2 via glycolysis restriction.* Nat Immunol, 2016. **17**(1): p. 95-103.

102. Rivadeneira, D.B. and G.M. Delgoffe, *Antitumor T-cell Reconditioning: Improving Metabolic Fitness for Optimal Cancer Immunotherapy.* Clin Cancer Res, 2018. **24**(11): p. 2473-2481.

103. Warburg, O., *On the origin of cancer cells.* Science (New York, N.Y.), 1956. **123**(3191): p. 309-314.

104. Reinfeld, B.I., et al., *Cell-programmed nutrient partitioning in the tumour microenvironment.* Nature, 2021. **593**(7858): p. 282-288.

105. Lee, H., et al., *Energy-stress-mediated AMPK activation inhibits ferroptosis.* Nature cell biology, 2020. **22**(2): p. 225-234.

106. Song, X., et al., *PDK4 dictates metabolic resistance to ferroptosis by suppressing pyruvate oxidation and fatty acid synthesis.* Cell Rep, 2021. **34**(8): p. 108767.

107. Cham, C.M., et al., *Glucose deprivation inhibits multiple key gene expression events and effector functions in CD8+ T cells.* Eur J Immunol, 2008. **38**(9): p. 2438-50.

108. Watson, M.J., et al., *Metabolic support of tumour-infiltrating regulatory T cells by lactic acid.* Nature, 2021. **591**(7851): p. 645-651.

109. Koppula, P., et al., *Amino acid transporter SLC7A11/xCT at the crossroads of regulating redox homeostasis and nutrient dependency of cancer.* Cancer communications (London, England), 2018. **38**(1): p. 12.

110. Kamphorst, J.J., et al., *Human pancreatic cancer tumors are nutrient poor and tumor cells actively scavenge extracellular protein.* Cancer research, 2015. **75**(3): p. 544-553.

111. Pan, M., et al., *Regional glutamine deficiency in tumours promotes dedifferentiation through inhibition of histone demethylation.* Nature cell biology, 2016. **18**(10): p. 1090-1101.

112. Hanahan, D. and R.A. Weinberg, *Hallmarks of cancer: the next generation.* Cell, 2011. **144**(5): p. 646-674.

113. Diehn, M., et al., *Association of reactive oxygen species levels and radioresistance in cancer stem cells.* Nature, 2009. **458**(7239): p. 780-783.

114. Cramer, S.L., et al., *Systemic depletion of L-cyst(e)ine with cyst(e)inase increases reactive oxygen species and suppresses tumor growth.* Nature medicine, 2017. **23**(1): p. 120-127.

115. Daher, B., et al., *Genetic Ablation of the Cystine Transporter xCT in PDAC Cells Inhibits mTORC1, Growth, Survival, and Tumor Formation via Nutrient and Oxidative Stresses.* Cancer research, 2019. **79**(15): p. 3877-3890.

116. Meira, W., et al., *A Cystine-Cysteine Intercellular Shuttle Prevents Ferroptosis in xCT Pancreatic Ductal Adenocarcinoma Cells.* Cancers, 2021. **13**(6).

117. Zhang, W., et al., *Stromal control of cystine metabolism promotes cancer cell survival in chronic lymphocytic leukaemia.* Nature cell biology, 2012. **14**(3): p. 276-286.

118. Zhang, Y., et al., *BAP1 links metabolic regulation of ferroptosis to tumour suppression.* Nature cell biology, 2018. **20**(10): p. 1181-1192.

119. Gao, M., et al., *Glutaminolysis and Transferrin Regulate Ferroptosis.* Molecular cell, 2015. **59**(2): p. 298-308.

120. Andrews, N.C. and P.J. Schmidt, *Iron homeostasis.* Annual review of physiology, 2007. **69**: p. 69-85.

121. Muir, A., et al., *Environmental cystine drives glutamine anaplerosis and sensitizes cancer cells to glutaminase inhibition.* eLife, 2017. **6**.

122. Cassago, A., et al., *Mitochondrial localization and structure-based phosphate activation mechanism of Glutaminase C with implications for cancer metabolism.* Proceedings of the National Academy of Sciences of the United States of America, 2012. **109**(4): p. 1092-1097.

123. Jennis, M., et al., *An African-specific polymorphism in the TP53 gene impairs p53 tumor suppressor function in a mouse model.* Genes & development, 2016. **30**(8): p. 918-930.

124. Bannai, S. and T. Ishii, *A novel function of glutamine in cell culture: utilization of glutamine for the uptake of cystine in human fibroblasts.* J Cell Physiol, 1988. **137**(2): p. 360-6.

125. MacIver, N.J., R.D. Michalek, and J.C. Rathmell, *Metabolic regulation of T lymphocytes.* Annu Rev Immunol, 2013. **31**: p. 259-83.

126. *CD36 Activity Causes Ferroptosis in Tumor-Infiltrating CD8 T Cells.* Cancer discovery, 2021. **11**(5): p. OF24.

127. García-Cañaveras, J.C., L. Chen, and J.D. Rabinowitz, *The Tumor Metabolic Microenvironment: Lessons from Lactate.* Cancer research, 2019. **79**(13): p. 3155-3162.

128. Ippolito, L., et al., *Lactate: A Metabolic Driver in the Tumour Landscape.* Trends in biochemical sciences, 2019. **44**(2): p. 153-166.

129. Manzo, T., et al., *Accumulation of long-chain fatty acids in the tumor microenvironment drives dysfunction in intrapancreatic CD8+ T cells.* The Journal of experimental medicine, 2020. **217**(8).

130. Ma, X., et al., *CD36-mediated ferroptosis dampens intratumoral CD8 T cell effector function and impairs their antitumor ability.* Cell metabolism, 2021.

131. Zhang, Y., et al., *Enhancing CD8 T Cell Fatty Acid Catabolism within a Metabolically Challenging Tumor Microenvironment Increases the Efficacy of Melanoma Immunotherapy.* Cancer cell, 2017. **32**(3).

132. Yang, W.S., et al., *Peroxidation of polyunsaturated fatty acids by lipoxygenases drives ferroptosis.* Proceedings of the National Academy of Sciences of the United States of America, 2016. **113**(34): p. E4966-E4975.

133. Gurusamy, D., et al., *Multi-phenotype CRISPR-Cas9 Screen Identifies p38 Kinase as a Target for Adoptive Immunotherapies.* Cancer cell, 2020. **37**(6).

134. Xu, S., et al., *Uptake of oxidized lipids by the scavenger receptor CD36 promotes lipid peroxidation and dysfunction in CD8 T cells in tumors.* Immunity, 2021. **54**(7).

135. Luo, X., et al., *The fatty acid receptor CD36 promotes HCC progression through activating Src/PI3K/AKT axis-dependent aerobic glycolysis.* Cell Death Dis, 2021. **12**(4): p. 328.

136. Liu, W., et al., *Dysregulated cholesterol homeostasis results in resistance to ferroptosis increasing tumorigenicity and metastasis in cancer.* Nat Commun, 2021. **12**(1): p. 5103.

137. Wang, Z.H., et al., *Lactate in the tumour microenvironment: From immune modulation to therapy.* EBioMedicine, 2021. **73**: p. 103627.

138. Zhao, Y., et al., *HCAR1/MCT1 Regulates Tumor Ferroptosis through the Lactate-Mediated AMPK-SCD1 Activity and Its Therapeutic Implications.* Cell reports, 2020. **33**(10): p. 108487.

139. Pereira, E.R., et al., *Lymph node metastases can invade local blood vessels, exit the node, and colonize distant organs in mice.* Science (New York, N.Y.), 2018. **359**(6382): p. 1403-1407.

140. Brown, M., et al., *Lymph node blood vessels provide exit routes for metastatic tumor cell dissemination in mice.* Science (New York, N.Y.), 2018. **359**(6382): p. 1408-1411.

141. Tang, Y., et al., *Fatty acid activation in carcinogenesis and cancer development: Essential roles of long-chain acyl-CoA synthetases.* Oncology letters, 2018. **16**(2): p. 1390-1396.

142. Ubellacker, J.M., et al., *Lymph protects metastasizing melanoma cells from ferroptosis.* Nature, 2020. **585**(7823): p. 113-118.

143. Piskounova, E., et al., *Oxidative stress inhibits distant metastasis by human melanoma cells.* Nature, 2015. **527**(7577): p. 186-191.

144. Le Gal, K., et al., *Antioxidants can increase melanoma metastasis in mice.* Science translational medicine, 2015. **7**(308): p. 308re8.

145. Li, Z., et al., *Carbonic anhydrase 9 confers resistance to ferroptosis/apoptosis in malignant mesothelioma under hypoxia.* Redox Biol, 2019. **26**: p. 101297.

146. Fan, Z., et al., *Hypoxia blocks ferroptosis of hepatocellular carcinoma via suppression of METTL14 triggered YTHDF2-dependent silencing of SLC7A11.* J Cell Mol Med, 2021. **25**(21): p. 10197-10212.

147. Xiong, J., et al., *Hypoxia Enhances HIF1α Transcription Activity by Upregulating KDM4A and Mediating H3K9me3, Thus Inducing Ferroptosis Resistance in Cervical Cancer Cells.* Stem Cells Int, 2022. **2022**: p. 1608806.

148. Lin, Z., et al., *Hypoxia-induced HIF-1α/lncRNA-PMAN inhibits ferroptosis by promoting the cytoplasmic translocation of ELAVL1 in peritoneal dissemination from gastric cancer.* Redox Biol, 2022. **52**: p. 102312.

149. Zou, Y., et al., *A GPX4-dependent cancer cell state underlies the clear-cell morphology and confers sensitivity to ferroptosis.* Nature communications, 2019. **10**(1): p. 1617.

150. Harris, A.L., *Hypoxia--a key regulatory factor in tumour growth.* Nature reviews. Cancer, 2002. **2**(1): p. 38-47.

151. Jing, X., et al., *Role of hypoxia in cancer therapy by regulating the tumor microenvironment.* Molecular cancer, 2019. **18**(1): p. 157.

152. Alvarez, S.W., et al., *NFS1 undergoes positive selection in lung tumours and protects cells from ferroptosis.* Nature, 2017. **551**(7682): p. 639-643.

153. Henze, A.-T. and M. Mazzone, *The impact of hypoxia on tumor-associated macrophages.* The Journal of clinical investigation, 2016. **126**(10): p. 3672-3679.

154. Soares, M.P. and I. Hamza, *Macrophages and Iron Metabolism.* Immunity, 2016. **44**(3): p. 492-504.

155. Fuhrmann, D.C., et al., *Hypoxia inhibits ferritinophagy, increases mitochondrial ferritin, and protects from ferroptosis.* Redox biology, 2020. **36**: p. 101670.

156. Gryzik, M., et al., *Expression and characterization of the ferritin binding domain of Nuclear Receptor Coactivator-4 (NCOA4).* Biochimica et biophysica acta. General subjects, 2017. **1861**(11 Pt A): p. 2710-2716.

157. Ajoolabady, A., et al., *Ferritinophagy and ferroptosis in the management of metabolic diseases.* Trends Endocrinol Metab, 2021. **32**(7): p. 444-462.

158. Ni, S., et al., *Hypoxia inhibits RANKL-induced ferritinophagy and protects osteoclasts from ferroptosis.* Free radical biology & medicine, 2021. **169**: p. 271-282.

159. Taylor, M., et al., *Hypoxia-inducible factor-2α mediates the adaptive increase of intestinal ferroportin during iron deficiency in mice.* Gastroenterology, 2011. **140**(7): p. 2044-2055.

160. Wu, J., et al., *Intercellular interaction dictates cancer cell ferroptosis via NF2-YAP signalling.* Nature, 2019. **572**(7769): p. 402-406.

161. Poursaitidis, I., et al., *Oncogene-Selective Sensitivity to Synchronous Cell Death following Modulation of the Amino Acid Nutrient Cystine.* Cell reports, 2017. **18**(11): p. 2547-2556.

162. Yang, W.-H., et al., *The Hippo Pathway Effector TAZ Regulates Ferroptosis in Renal Cell Carcinoma.* Cell reports, 2019. **28**(10).

163. Yang, W.-H., et al., *A TAZ-ANGPTL4-NOX2 Axis Regulates Ferroptotic Cell Death and Chemoresistance in Epithelial Ovarian Cancer.* Molecular cancer research : MCR, 2020. **18**(1): p. 79-90.

164. Alizadeh, A.A., et al., *Toward understanding and exploiting tumor heterogeneity.* Nature medicine, 2015. **21**(8): p. 846-853.

165. McGranahan, N. and C. Swanton, *Clonal Heterogeneity and Tumor Evolution: Past, Present, and the Future.* Cell, 2017. **168**(4): p. 613-628.

166. Quezada, S.A., et al., *CTLA4 blockade and GM-CSF combination immunotherapy alters the intratumor balance of effector and regulatory T cells.* The Journal of clinical investigation, 2006. **116**(7): p. 1935-1945.

167. Galon, J., et al., *Type, density, and location of immune cells within human colorectal tumors predict clinical outcome.* Science (New York, N.Y.), 2006. **313**(5795): p. 1960-1964.

168. Galon, J. and D. Bruni, *Approaches to treat immune hot, altered and cold tumours with combination immunotherapies.* Nature reviews. Drug discovery, 2019. **18**(3): p. 197-218.

169. Sharma, P., et al., *Primary, Adaptive, and Acquired Resistance to Cancer Immunotherapy.* Cell, 2017. **168**(4): p. 707-723.

170. De Henau, O., et al., *Overcoming resistance to checkpoint blockade therapy by targeting PI3Kγ in myeloid cells.* Nature, 2016. **539**(7629): p. 443-447.

171. Ren, D., et al., *Predictive biomarkers and mechanisms underlying resistance to PD1/PD-L1 blockade cancer immunotherapy.* Molecular cancer, 2020. **19**(1): p. 19.

172. Tsoi, J., et al., *Multi-stage Differentiation Defines Melanoma Subtypes with Differential Vulnerability to Drug-Induced Iron-Dependent Oxidative Stress.* Cancer cell, 2018. **33**(5).

173. Lee, J., et al., *Epigenetic reprogramming of epithelial-mesenchymal transition promotes ferroptosis of head and neck cancer.* Redox biology, 2020. **37**: p. 101697.

174. He, Y., et al., *Multi-omics characterization and therapeutic liability of ferroptosis in melanoma.* Signal Transduct Target Ther, 2022. **7**(1): p. 268.

175. Wan, R.J., et al., *Ferroptosis-related gene signature predicts prognosis and immunotherapy in glioma.* CNS Neurosci Ther, 2021. **27**(8): p. 973-986.

176. Duan, Q., et al., *Turning Cold into Hot: Firing up the Tumor Microenvironment.* Trends in cancer, 2020. **6**(7): p. 605-618.

177. Yang, L., et al., *PKM2 regulates the Warburg effect and promotes HMGB1 release in sepsis.* Nature communications, 2014. **5**: p. 4436.

178. Camus, M., et al., *Coordination of intratumoral immune reaction and human colorectal cancer recurrence.* Cancer research, 2009. **69**(6): p. 2685-2693.

179. Mlecnik, B., et al., *Functional network pipeline reveals genetic determinants associated with in situ lymphocyte proliferation and survival of cancer patients.* Science translational medicine, 2014. **6**(228): p. 228ra37.

180. Spranger, S., et al., *Tumor-Residing Batf3 Dendritic Cells Are Required for Effector T Cell Trafficking and Adoptive T Cell Therapy.* Cancer cell, 2017. **31**(5).

181. Mariathasan, S., et al., *TGFβ attenuates tumour response to PD-L1 blockade by contributing to exclusion of T cells.* Nature, 2018. **554**(7693): p. 544-548.

182. van der Woude, L.L., et al., *Migrating into the Tumor: a Roadmap for T Cells.* Trends Cancer, 2017. **3**(11): p. 797-808.

183. Tan, S.K., et al., *Obesity-Dependent Adipokine Chemerin Suppresses Fatty Acid Oxidation to Confer Ferroptosis Resistance.* Cancer Discov, 2021. **11**(8): p. 2072-2093.

184. Zhu, J., et al., *Transsulfuration Activity Can Support Cell Growth upon Extracellular Cysteine Limitation.* Cell metabolism, 2019. **30**(5).

185. Erdélyi, K., et al., *Reprogrammed transsulfuration promotes basal-like breast tumor progression via realigning cellular cysteine persulfidation.* Proc Natl Acad Sci U S A, 2021. **118**(45).

186. Yang, F., et al., *Ferroptosis heterogeneity in triple-negative breast cancer reveals an innovative immunotherapy combination strategy.* Cell Metab, 2023. **35**(1): p. 84-100.e8.

187. Bebber, C.M., et al., *Ferroptosis response segregates small cell lung cancer (SCLC) neuroendocrine subtypes.* Nat Commun, 2021. **12**(1): p. 2048.

188. Goji, T., et al., *Cystine uptake through the cystine/glutamate antiporter xCT triggers glioblastoma cell death under glucose deprivation.* The Journal of biological chemistry, 2017. **292**(48): p. 19721-19732.

189. Shin, C.-S., et al., *The glutamate/cystine xCT antiporter antagonizes glutamine metabolism and reduces nutrient flexibility.* Nature communications, 2017. **8**: p. 15074.

190. Leone, R.D., et al., *Glutamine blockade induces divergent metabolic programs to overcome tumor immune evasion.* Science (New York, N.Y.), 2019. **366**(6468): p. 1013-1021.

191. Jones, S., et al., *Core signaling pathways in human pancreatic cancers revealed by global genomic analyses.* Science (New York, N.Y.), 2008. **321**(5897): p. 1801-1806.

192. Commisso, C., et al., *Macropinocytosis of protein is an amino acid supply route in Ras-transformed cells.* Nature, 2013. **497**(7451): p. 633-637.

193. Wise, D.R., et al., *Myc regulates a transcriptional program that stimulates mitochondrial glutaminolysis and leads to glutamine addiction.* Proceedings of the National Academy of Sciences of the United States of America, 2008. **105**(48): p. 18782-18787.

194. Corbet, C. and O. Feron, *Tumour acidosis: from the passenger to the driver's seat.* Nat Rev Cancer, 2017. **17**(10): p. 577-593.

195. Rey, E., et al., *Hypoxia-inducible factor 2α drives hepatosteatosis through the fatty acid translocase CD36.* Liver Int, 2020. **40**(10): p. 2553-2567.

196. Mwaikambo, B.R., et al., *Hypoxia up-regulates CD36 expression and function via hypoxia-inducible factor-1- and phosphatidylinositol 3-kinase-dependent mechanisms.* J Biol Chem, 2009. **284**(39): p. 26695-707.

197. Ino, Y., et al., *Immune cell infiltration as an indicator of the immune microenvironment of pancreatic cancer.* British journal of cancer, 2013. **108**(4): p. 914-923.

198. Liu, T., et al., *Ferroptosis, as the most enriched programmed cell death process in glioma, induces immunosuppression and immunotherapy resistance.* Neuro Oncol, 2022. **24**(7): p. 1113-1125.

199. Fan, F., et al., *A Dual PI3K/HDAC Inhibitor Induces Immunogenic Ferroptosis to Potentiate Cancer Immune Checkpoint Therapy.* Cancer Res, 2021. **81**(24): p. 6233-6245.

200. Mao, W., et al., *Statin shapes inflamed tumor microenvironment and enhances immune checkpoint blockade in non-small cell lung cancer.* JCI Insight, 2022. **7**(18).

201. Cheu, J.W., et al., *Ferroptosis Suppressor Protein 1 Inhibition Promotes Tumor Ferroptosis and Anti-tumor Immune Responses in Liver Cancer.* Cell Mol Gastroenterol Hepatol, 2023. **16**(1): p. 133-159.

202. Ruan, W.Y., et al., *An inflammation-associated ferroptosis signature optimizes the diagnosis, prognosis evaluation and immunotherapy options in hepatocellular carcinoma.* J Cell Mol Med, 2023. **27**(13): p. 1820-1835.

203. Xue, Y., et al., *Intermittent dietary methionine deprivation facilitates tumoral ferroptosis and synergizes with checkpoint blockade.* Nat Commun, 2023. **14**(1): p. 4758.

204. Li, H., et al., *Ferritin light chain promotes the reprogramming of glioma immune microenvironment and facilitates glioma progression.* Theranostics, 2023. **13**(11): p. 3794-3813.

205. Siegel, R.L., et al., *Cancer Statistics, 2021.* CA: a cancer journal for clinicians, 2021. **71**(1).

206. Yu, W., et al., *Acquisition of Cisplatin Resistance Shifts Head and Neck Squamous Cell Carcinoma Metabolism toward Neutralization of Oxidative Stress.* Cancers, 2020. **12**(6).

207. Galluzzi, L., et al., *Molecular mechanisms of cisplatin resistance.* Oncogene, 2012. **31**(15): p. 1869-1883.

208. Zhu, H., et al., *Molecular mechanisms of cisplatin resistance in cervical cancer.* Drug design, development and therapy, 2016. **10**: p. 1885-1895.

209. Wang, G.X., et al., *ΔNp63 Inhibits Oxidative Stress-Induced Cell Death, Including Ferroptosis, and Cooperates with the BCL-2 Family to Promote Clonogenic Survival.* Cell reports, 2017. **21**(10): p. 2926-2939.

210. Wang, Y., et al., *Frizzled-7 Identifies Platinum-Tolerant Ovarian Cancer Cells Susceptible to Ferroptosis.* Cancer research, 2021. **81**(2): p. 384-399.

211. Okuno, S., et al., *Role of cystine transport in intracellular glutathione level and cisplatin resistance in human ovarian cancer cell lines.* British journal of cancer, 2003. **88**(6): p. 951-956.

212. Huang, Y., et al., *Cystine-glutamate transporter SLC7A11 in cancer chemosensitivity and chemoresistance.* Cancer research, 2005. **65**(16): p. 7446-7454.

213. Chen, H.H.W. and M.T. Kuo, *Role of glutathione in the regulation of Cisplatin resistance in cancer chemotherapy.* Metal-based drugs, 2010. **2010**.

214. Guo, J., et al., *Ferroptosis: A Novel Anti-tumor Action for Cisplatin.* Cancer research and treatment, 2018. **50**(2): p. 445-460.

215. Song, Z., et al., *Exosomal miR-4443 promotes cisplatin resistance in non-small cell lung carcinoma by regulating FSP1 m6A modification-mediated ferroptosis.* Life sciences, 2021. **276**: p. 119399.

216. Sato, M., et al., *The ferroptosis inducer erastin irreversibly inhibits system x- and synergizes with cisplatin to increase cisplatin's cytotoxicity in cancer cells.* Scientific reports, 2018. **8**(1): p. 968.

217. Roh, J.-L., et al., *Induction of ferroptotic cell death for overcoming cisplatin resistance of head and neck cancer.* Cancer letters, 2016. **381**(1).

218. Hu, Z., et al., *A Potential Mechanism of Temozolomide Resistance in Glioma-Ferroptosis.* Frontiers in oncology, 2020. **10**: p. 897.

219. Chen, L., et al., *Erastin sensitizes glioblastoma cells to temozolomide by restraining xCT and cystathionine-γ-lyase function.* Oncology reports, 2015. **33**(3): p. 1465-1474.

220. Chaudhary, N., et al., *Lipocalin 2 expression promotes tumor progression and therapy resistance by inhibiting ferroptosis in colorectal cancer.* International journal of cancer, 2021. **149**(7): p. 1495-1511.

221. Bi, J., et al., *Metadherin enhances vulnerability of cancer cells to ferroptosis.* Cell death & disease, 2019. **10**(10): p. 682.

222. Strobel, O., et al., *Optimizing the outcomes of pancreatic cancer surgery.* Nature reviews. Clinical oncology, 2019. **16**(1): p. 11-26.

223. Tang, R., et al., *The role of ferroptosis regulators in the prognosis, immune activity and gemcitabine resistance of pancreatic cancer.* Annals of translational medicine, 2020. **8**(21): p. 1347.

224. Lo, M., et al., *The xc- cystine/glutamate antiporter: a mediator of pancreatic cancer growth with a role in drug resistance.* British journal of cancer, 2008. **99**(3): p. 464-472.

225. Zhang, Z., et al., *Holo-lactoferrin: the link between ferroptosis and radiotherapy in triple-negative breast cancer.* Theranostics, 2021. **11**(7): p. 3167-3182.

226. Lei, G., et al., *The role of ferroptosis in ionizing radiation-induced cell death and tumor suppression.* Cell research, 2020. **30**(2): p. 146-162.

227. Ye, L.F., et al., *Radiation-Induced Lipid Peroxidation Triggers Ferroptosis and Synergizes with Ferroptosis Inducers.* ACS chemical biology, 2020. **15**(2): p. 469-484.

228. Tomita, K., et al., *MiR-7-5p Is Involved in Ferroptosis Signaling and Radioresistance Thru the Generation of ROS in Radioresistant HeLa and SAS Cell Lines.* Int J Mol Sci, 2021. **22**(15).

229. Koppula, P., et al., *A targetable CoQ-FSP1 axis drives ferroptosis- and radiation-resistance in KEAP1 inactive lung cancers.* Nat Commun, 2022. **13**(1): p. 2206.

230. Lei, G., et al., *Ferroptosis as a mechanism to mediate p53 function in tumor radiosensitivity.* Oncogene, 2021. **40**(20): p. 3533-3547.

231. Wan, C., et al., *Irradiated tumor cell-derived microparticles mediate tumor eradication via cell killing and immune reprogramming.* Science advances, 2020. **6**(13): p. eaay9789.

232. Lang, X., et al., *Radiotherapy and Immunotherapy Promote Tumoral Lipid Oxidation and Ferroptosis via Synergistic Repression of SLC7A11.* Cancer discovery, 2019. **9**(12): p. 1673-1685.

233. Chen, P.-H., et al., *Kinome screen of ferroptosis reveals a novel role of ATM in regulating iron metabolism.* Cell death and differentiation, 2020. **27**(3): p. 1008-1022.

234. Shibata, Y., et al., *Erastin, a ferroptosis-inducing agent, sensitized cancer cells to X-ray irradiation via glutathione starvation in vitro and in vivo.* PloS one, 2019. **14**(12): p. e0225931.

235. Bertout, J.A., S.A. Patel, and M.C. Simon, *The impact of O2 availability on human cancer.* Nat Rev Cancer, 2008. **8**(12): p. 967-75.

236. Friedmann Angeli, J.P., D.V. Krysko, and M. Conrad, *Ferroptosis at the crossroads of cancer-acquired drug resistance and immune evasion.* Nature reviews. Cancer, 2019. **19**(7): p. 405-414.

237. Brown, C.W., et al., *Prominin2 Drives Ferroptosis Resistance by Stimulating Iron Export.* Developmental cell, 2019. **51**(5).

238. Chatterjee, N. and T.G. Bivona, *Polytherapy and Targeted Cancer Drug Resistance.* Trends in cancer, 2019. **5**(3): p. 170-182.

239. Louandre, C., et al., *Iron-dependent cell death of hepatocellular carcinoma cells exposed to sorafenib.* Int J Cancer, 2013. **133**(7): p. 1732-42.

240. Min, Y., et al., *Combating the drug resistance of cisplatin using a platinum prodrug based delivery system.* Angew Chem Int Ed Engl, 2012. **51**(27): p. 6742-7.

241. Eling, N., et al., *Identification of artesunate as a specific activator of ferroptosis in pancreatic cancer cells.* Oncoscience, 2015. **2**(5): p. 517-32.

242. Xu, H., et al., *Ferroptosis in the tumor microenvironment: perspectives for immunotherapy.* Trends Mol Med, 2021. **27**(9): p. 856-867.

243. Whiteside, T.L., et al., *Emerging Opportunities and Challenges in Cancer Immunotherapy.* Clin Cancer Res, 2016. **22**(8): p. 1845-55.

244. McLaughlin, M., et al., *Inflammatory microenvironment remodelling by tumour cells after radiotherapy.* Nature reviews. Cancer, 2020. **20**(4): p. 203-217.

245. Angelin, A., et al., *Foxp3 Reprograms T Cell Metabolism to Function in Low-Glucose, High-Lactate Environments.* Cell Metab, 2017. **25**(6): p. 1282-1293.e7.

**
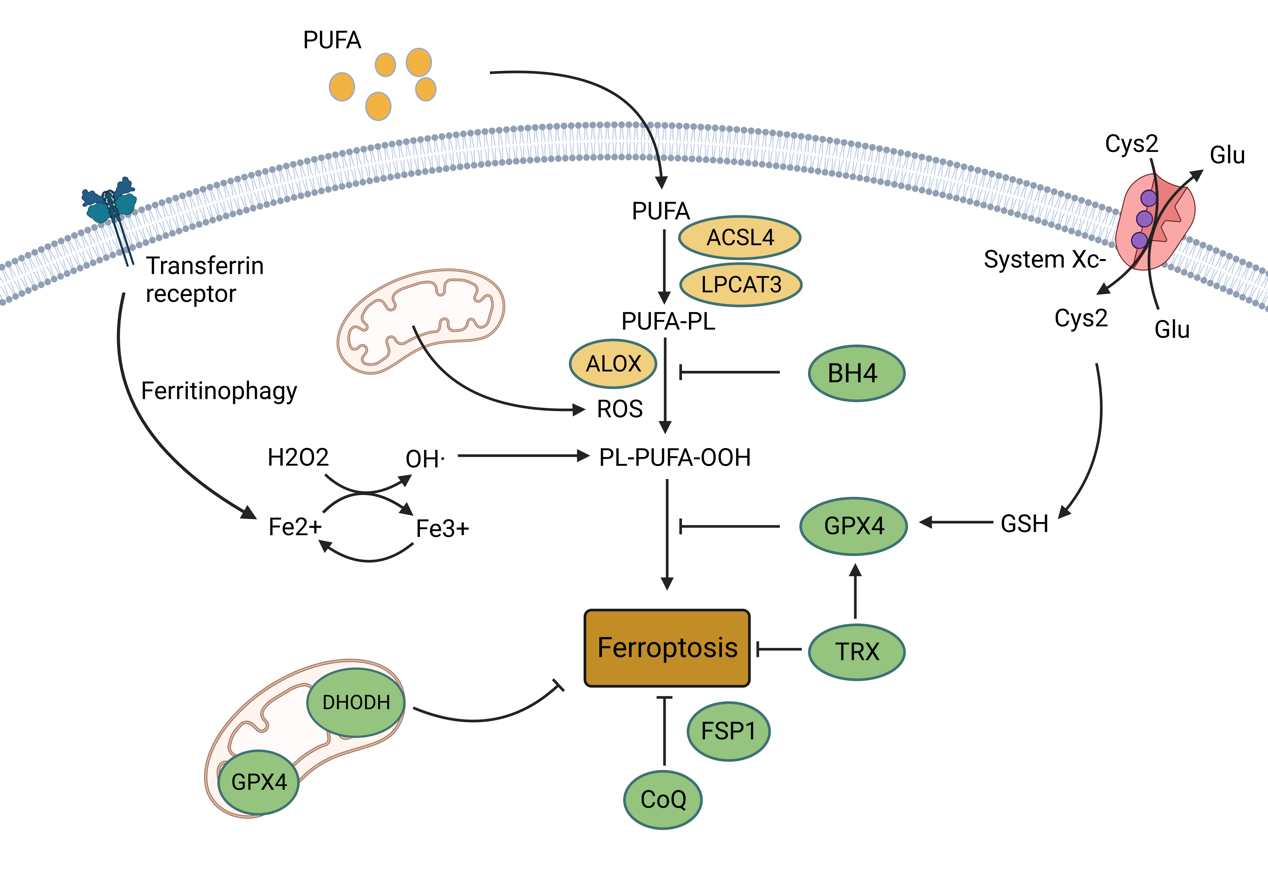
**

**Figure 1.** Regulation of ferroptosis [242]: cells require PUFAs for maintaining energy metabolism, with tremendous ROS production to oxidize PUFAs, especially AAs into PLOOH, which leads to cell membrane rupture and cell death. Iron catalyzes this process. System Xc^-^ transports Cys_2_ for the synthesis of GSH. GPX4 reduces the PLOOH into PLOH by GSH to resist ferroptosis. TXN pathway plays as an alternative way when GPX4 is inhibited. BH4 and CoQ also act as anti-ferroptosis way due to the function of eliminating ROS. In mitochondria, where ROS proliferates, DHODH inhibits mitochondrial ferroptosis cooperating with mitochondria GPX4.

**
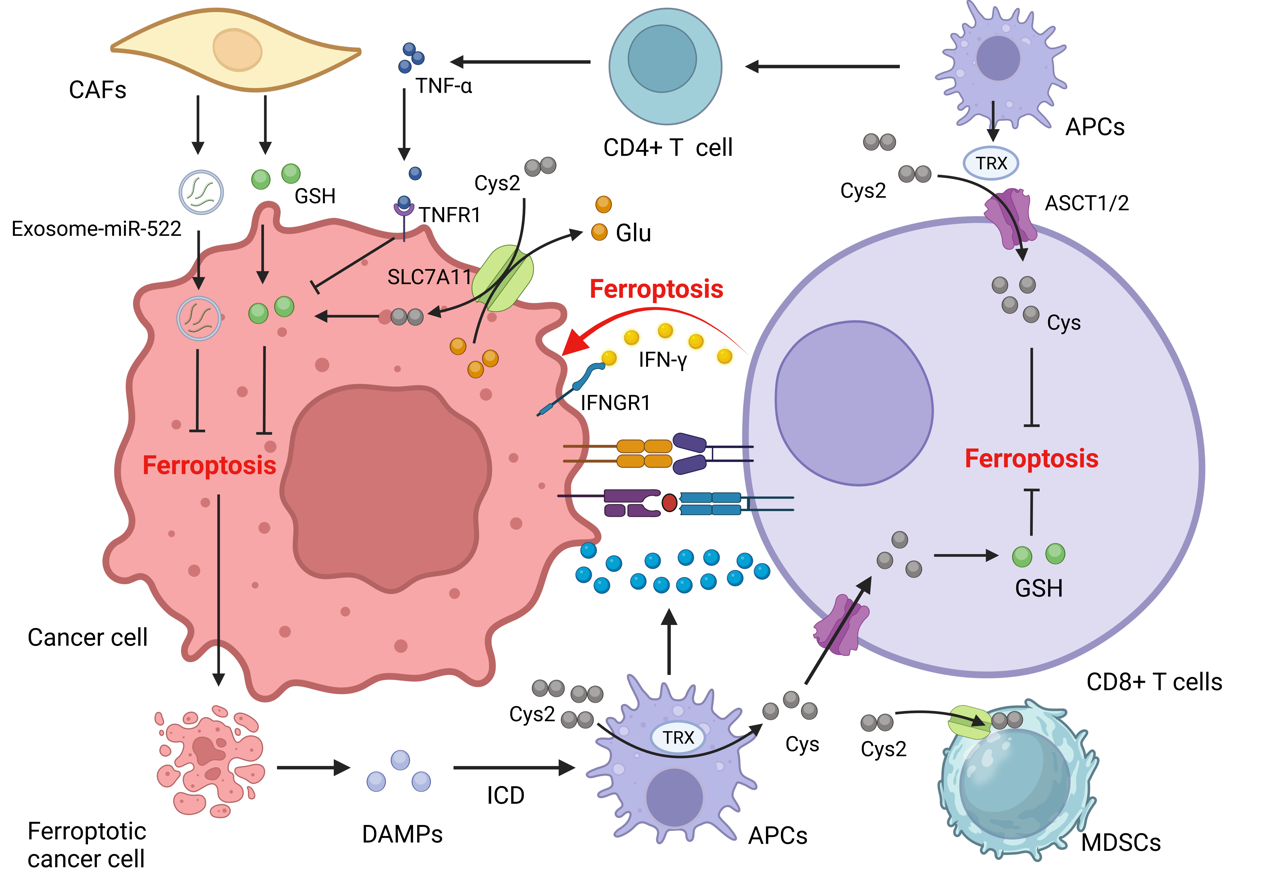
**

**Figure 2.** The complex interaction of cells in the TME: CD8^+^ T cells kill cancer cells by promoting ferroptosis. Ferroptotic cancer cells release damage-associated molecular patterns (DAMPs), inducing the maturation of DCs and enhancing immune response. Activated APCs like DCs and macrophages reduce Cys_2_ into Cys to supply CD8^+^ T cells for GSH synthesis. MDSCs with a powerful intracellular storage capacity of Cys_2_ compete with APCs for Cys_2_, promoting T cell ferroptosis. CAFs are principal donor cells of GSH for cancer cells to help cancer cells fight ferroptosis and resist chemotherapy drugs like Pt. CAFs also secrete exosome-miR-522 to inhibit ALOX15 translation in cancer cells. CD8^+^ T cells promote GGT5 and interfere with system Xc^-^ expression reducing GSH supplements for cancer cells.

**
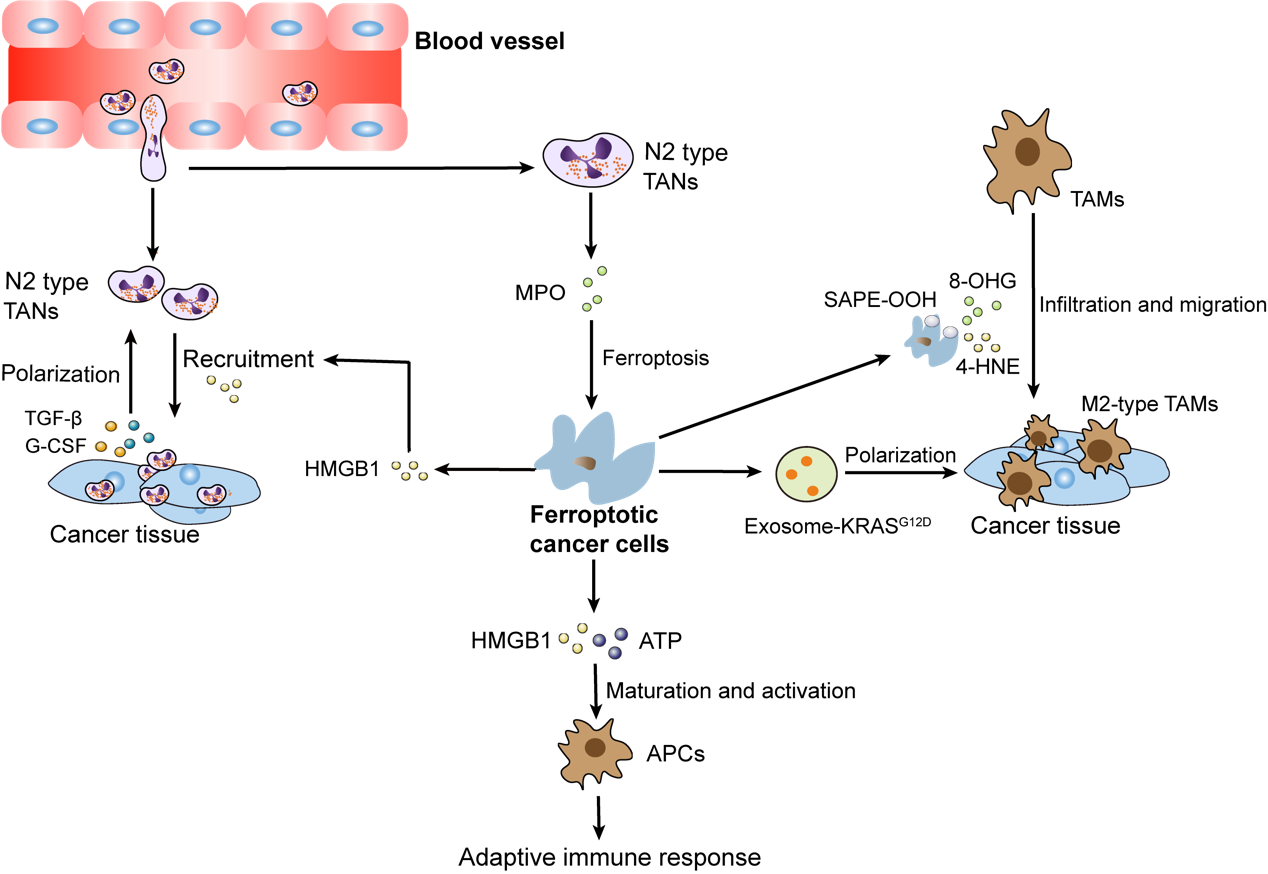
**

**Figure 3.** TANs are attracted by necrosis cancer tissue releasing HMGB1 and move to TME, where they are polarized and activated as N2-type TANs by TGF-β and G-CSF secreted from cancer cells. These TANs promote the ferroptosis of cancer cells by MPO, which fuels tumor progression. Ferroptotic cancer cells attract TAMs by other DAMPs, including 4-HNE, 8-OHG, and SAPE-OOH, and these TAMs are polarized into pro-tumor M2-type TAMs. Ferroptotic cancer cells amplify immune response by inducing ICD to mature and activate APCs.

**
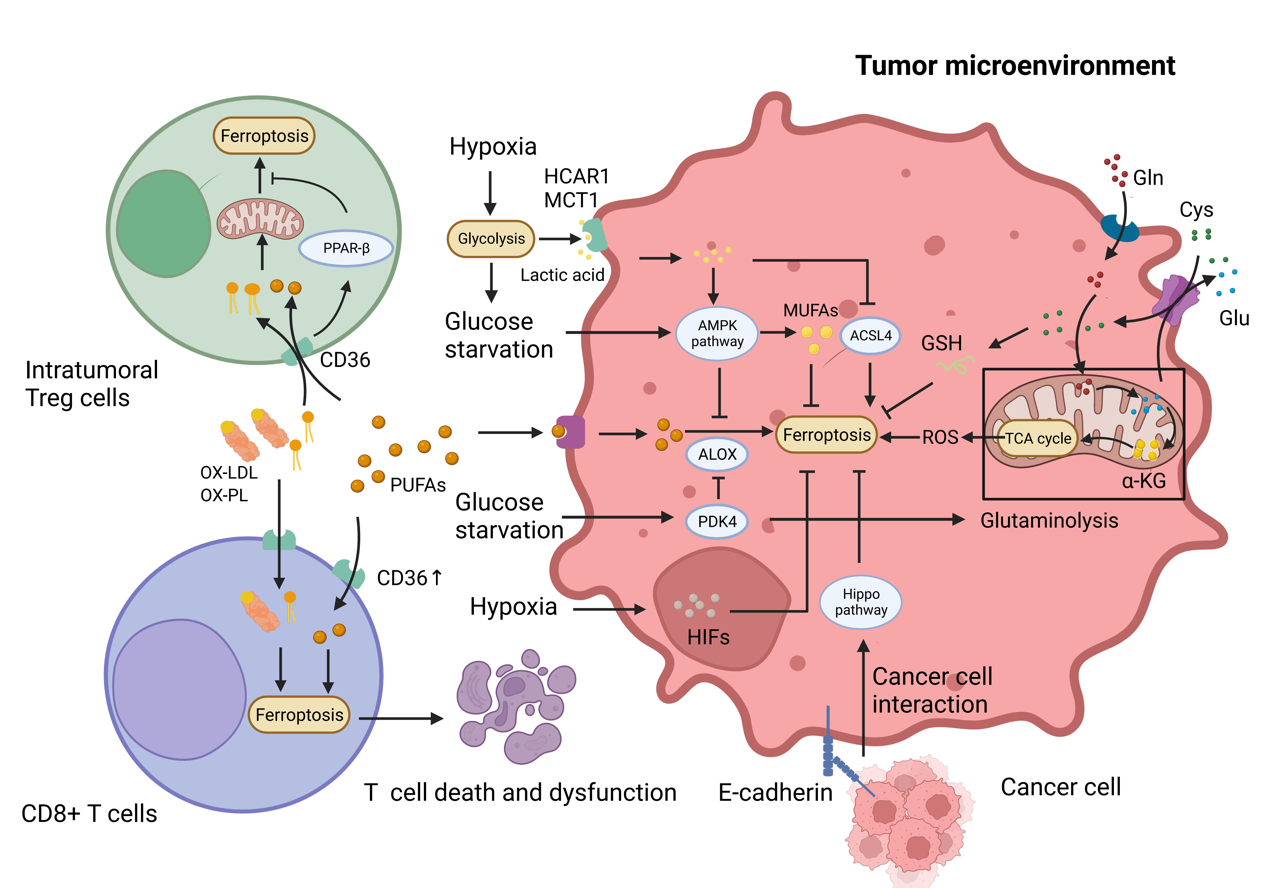
**

**Figure 4.** Various metabolism process exists in TME, including lipid, amino acid, and glucose metabolism. Cholesterol in TME induces CD36 overexpression in T cells to promote the absorption of PUFAs, Ox-LDL, and Ox-PL in cells, fueling the ferroptosis of Tregs and CD8^+^ T cells. However, CD36 expression on Tregs maintains mitochondrial fitness to protect them from ferroptosis. Intensive glycolysis of cancer cells in TME leads to glucose starvation. It dictates ferroptosis resistance by activating the AMPK pathway to improve the MUFAs proportion of cell membrane and inducing over-expression of PDK4 to repress the TCA cycle and activity of ALOX. Lactic acid is taken in cancer cells and activates the AMPK pathway and ACSL4 to inhibit ferroptosis. Gln has recently been thought of as the leading energy metabolism resource participating in TCA and FA synthesis by glutaminolysis. Gln metabolism antagonizes with system Xc^-^ antioxidation function.

**Table 1.** Possible combination therapy for these immunophenotypes of TME with different immune feature

| Immune phenotype | Features | Treatment strategy |
| --- | --- | --- |
| Cold tumor | T cell deficiency, low tumor mutational burden, and poor antigen presentation [168] | Promote cancer ferroptosis to heat cancer and combine with immunotherapy like ICB. |
| Immune-suppressive tumor  Immune-excluded tumor  Hot tumor | Full of immunosuppressive cells like MDSCs, Tregs, and M2-type TAMs with local adaptive immunosuppression [243]  ECM, hypoxia, and deficient chemokines inhibiting the recruitment of T cells and establishing immunosuppression [168]  Adequate infiltration of anti-cancer T cells in the center of TME [244] | Combine immunotherapy with ferroptosis therapy targeting immunosuppressive cells, especially MDSCs, and M2-TAMs.  Need more investigation. HIFs inhibitors combined with ferroptosis therapy and chemokines may be an optional method.  Combine with ferroptosis therapy when drug resistance to immunotherapy occurs, especially for cancer cells with dedifferentiated phenotypes. |

**Table 2.** The possible microenvironmental sensitization biomarkers and targets synergistic with ferroptosis inducers and sensitizing mechanisms

| Synergistic sensitization targets | Microenvironmental factors | Mechanisms |
| --- | --- | --- |
| CD36 and HIFs | Lipids, PUFAs, and  hypoxia | Suppression of CD36 expression decreases ferroptosis sensitivity of anti-tumor T cells and destroys mitochondria fitness of Treg. HIFs antibody prevents pro-tumor polarization of TAMs and facilitates M2-type TAMs ferroptosis by affecting iron metabolism [153, 154]. CD36 and HIFs have a synergistic effect [195, 196]. |
| HCAR1and MCT1  Gln/system Xc^-^  E-cadherin | Lactic acid  Glucose, Gln and  Cys  Cancer cell  interaction | The abundant lactic acid in the TME is taken in cancer cells by transporters HCAR1 and MCT1. It activates the AMPK pathway to produce more anti-ferroptosis MUFAs, and a high level of lactic acid also inhibits the expression of ACSL4 [138, 245].  Gln metabolism antagonizes the antioxidation function of system Xc^-^. Cys-deprivation triggers the death of cancer cells with extensive expression of system Xc^-^. Conversely, CDI ferroptosis occurs in cancer cells with low expression of system Xc^-^ [109].  Interaction of cancer cells promotes the expression of E-cadherin to activate the Hippo pathway, inhibiting the expression of ANGPTL4, TFRC, and ACSL4 and the production of ROS to maintain resistance to ferroptosis [160, 162]. |
