# Supplementary material for: Modulating ferroptosis sensitivity: environmental and cellular targets within the tumor microenvironment
Source: J Exp Clin Cancer Res. 2024 Jan 13;43:19. doi: 10.1186/s13046-023-02925-5 (PMC10787430; doi:10.1186/s13046-023-02925-5)
Supplement: Supplementary file 2 — Supplementary Material 2 [file 13046_2023_2925_MOESM2_ESM.docx]

Dear Editor,

Thank you for giving us the opportunity to revise our submission. We carefully read all the comments from the reviewers and then revised the manuscript according to the comments. Thank all the reviewers for their valuable comments. Their comments are greatly helpful and constructive for improving the quality of this manuscript. Herein, we presented our reply to all the comments in a point-to-point way.

Reviewer #1: Manuscript entitled "Modulating Ferroptosis Sensitivity: Environmental and Cellular Targets within the Tumor Microenvironment " offers a comprehensive overview of ferroptosis and its interaction with the immune microenvironment. It delves into the intricate mutual regulation of ferroptosis between cancer cells and immune cells. Furthermore, it introduces how factors within the tumor microenvironment influence the sensitivity to ferroptosis. Finally, the article examines the potential of treating tumors by combining ferroptosis inducers with metabolic regulators. While the article provides valuable insights into the relationship between ferroptosis and immunity, I have a couple of concerns: 

1.The article delves into the relationship between ferroptosis and radiotherapy/chemotherapy, yet this content isn't directly related to immunity, it would be beneficial to further explore its implications for immunity.

**Reply: Thank you for your comment. We summarized the current progress in the use of ferroptosis in combination with immunotherapy. In this revision, we discussed it in the “6.1 Immunotherapy treatment with Ferroptosis in clinical” part which was highlighted in color.**

**“In the LAR subtype of TNBC, GPX4 inhibitors enhance the anti-tumor immunity. The combination of GPX4 inhibitor and PD-1 blockade is more efficient than monotherapy in vivo [199]. In glioma, in which ferroptosis is the main programmed cell death (PCD), ferroptosis contributed to immunosuppressive TME mediated by TAMs, which leads to malignant progression and poor proognnosis. Blocking ferroptosis is thought to be an efficient method to improve the efficacy of ICB [200]. Researchers in melanoma developed a ferroptosis score (FPS) model based on 32 ferroptosis-related genes to predict the outcome of cancer. Then, they found that high-FPS may predict more active tumor immune microenvironment and better efficacy of ICB such as PD-1 blockade [177]. Fan et al. found that BEBT-908, an inhibitor targeting PI3K and HDAC, induced immunogenic ferroptotic cell death of cancer cells and enhanced ICB therapy by upregulating MHC class I and activating IFN-γ signal [201]. Statin promotes immunotherapy by promoting ferroptosis of NSCLC cancer cells and inhibiting PD-L1 expression [202]. FSP1 inhibition greatly promoted ferroptosis of cancer cells and infiltration of immune cells like DCs, macrophages, and T cells. iFSP1 synergistic with immunotherapy significantly reduced the burden of HCC in vivo [203]. Researchers found inflammation-associated ferroptosis (IAF) biomarkers positively predicts the PD-L1 expression, MSI, TMB and ICB response rate [204]. Short-time starvation of methionine promotes ferroptosis by stimulation of CHAC1 transcription synergistically with CD8+ T cells and enhances ICB therapy. Exogenous Methionine intermittent starvation, system xc- and PD-1 blockade combination treatment have a profound anti-tumor efficacy [205]. Ferritin light chain (FTL) facilitates cancer cell ferroptosis in GBM to polarize TAMs into the M2-type contributing to pro-tumor TME. Inhibition of FTL is a promising strategy to sensitize GBM to PD-1 blockade [206].**

2.The figures included in the article are not so impressive, consider enhancing the visual elements to make the figures more engaging and illustrative for readers.

**Reply: Thank you for your comment. we carefully modified and redrew these figures to make them concise, engaging, and illustrative for readers.**

3.The last part is too lengthy and the content is not concise enough. An appropriate summary is recommended.

**Reply: Thank you for your suggestion. In this revision, we summarized the last part into 2 paragraphs including our finding of therapeutic paradigm and its inherent challenges.**

Reviewer #2: Dear Authors,

Your manuscript on the role of ferroptosis in the tumor microenvironment is a comprehensive exploration of this complex subject area and offers insightful conclusions. The manuscript is well-structured, and the level of detail in your discussion is commendable. However, there are several areas that could benefit from revision. 
Areas of concern and errors: 

Several sections of your manuscript are quite dense and could benefit from additional clarification or simplification.

**Reply: Thank you for your advice. We have simplified the content of our sections, especially the third part and the fourth part, to make them read more concisely and clearly.**

Your manuscript could benefit from a more thorough introduction to the concept of ferroptosis for readers who may not be familiar with this area of research.

**Reply:Thank you for your advice. We've introduced ferroptosis in more details and redrawn the figure of ferroptosis mechanism (figure 1) to make them more precise and concise for readers。**

I noticed some issues related to the clarity and precision of your language, which may contribute to potential misunderstandings. 
**Reply: Thank you for your advice. In this revision, we checked the language of the whole review carefully and revised some potentially ambiguous sentences.**

In some parts of the manuscript, the connection between ferroptosis and the specific aspects of the tumor microenvironment discussed could be more explicitly stated. 
**Reply: Thanks for your comments. We revised the contents from the section 3 and section 4 on cellular and environmental interaction of ferroptosis in our manuscript to make the illustration more explicit.**

Recommendations: 

I recommend that you aim for a more concise and direct writing style to improve readability and understanding. 

It would be beneficial to explain more clearly the relationship between ferroptosis and the various aspects of the tumor microenvironment discussed in the manuscript. 

Proofreading the manuscript for grammatical errors and awkward phrasing would greatly enhance its readability. 

Your manuscript provides a comprehensive review of the role of ferroptosis in the tumor microenvironment. However, there are some areas that could benefit from revisions to enhance clarity and readability. 

Introduction - The introduction is a bit dense with information. It would be useful to simplify some of the language here to make it more accessible to a wider audience. For example, the sentence "Within the TME, cancer cells coexist with various immunosuppressive entities, including myeloid-derived suppressor cells (MDSCs), Tregs, and M2-type tumor-associated macrophages (TAMs)" could be simplified to "In the tumor microenvironment, cancer cells interact with various immune cells such as MDSCs, Tregs, and M2-type TAMs".

Paragraph 3.4 - The phrase "The ability of NO to freely traverse cellular membranes renders it effective in extending its protective shield against ferroptosis to neighboring cells [69, 70]." could be rewritten for clarity. Consider "The ability of NO to move freely across cellular membranes allows it to protect neighbouring cells from ferroptosis [69, 70]." 

Paragraph 3.5 - The sentence "This, coupled with their reluctance to re-export Cys into the TME, creates a scenario where MDSCs significantly deplete the extracellular pool of Cys2." is a bit unclear. A possible revision could be "Their reluctance to export Cys back into the TME, coupled with their active uptake of Cys2, results in a significant reduction in the extracellular pool of Cys2." √

Paragraph 6.2 - The sentence "Intrinsic attributes of the TME, such as hypoxia, can erect barriers to both radiotherapy and ferroptosis, orchestrated by the induction of hypoxia-inducible factors (HIFs) [250]." could be clarified. Consider revising to "Hypoxia in the TME can induce hypoxia-inducible factors (HIFs), creating barriers to both radiotherapy and ferroptosis [250]."

Conclusions and Perspectives - This section could benefit from a more concise summary of the main findings and how they contribute to the field. For example, the sentence "The clinical arena grapples with a dearth of efficacious and safe therapeutics targeting ferroptosis" could be simplified to "There is a need for more efficient and safe treatments targeting ferroptosis."

Thanks for your advice.

Please consider these suggestions when revising your manuscript. They are intended to enhance the readability and clarity of your paper, making it more accessible to a broader audience.

**Reply: Thanks for your recommendations. Based on the reviewers' valuable suggestions, we optimized our writing in a more precise and clear way. Moreover, the manuscript of the section on ferroptosis and TME interactions has been revised again to make their relationship more summarized and explicit. we carefully checked and revised the inappropriate writing, spelling, and grammar and We have all revised the examples listed above.**

We are confident that these revisions and the professional language editing have significantly enhanced the quality of our manuscript. We are thankful for the opportunity to refine our work and for the valuable feedback provided by the reviewers and the editorial team.

Sincerely

Quan Liao, M.D.

Professor, Department of General Surgery

Peking Union Medical College Hospital

Peking Union Medical College and Chinese Academy of Medical Sciences

Beijing, China 100730
